# Nano-enabled disruption of bacterial virulence and communication in plant pathosystems: emerging strategies for sustainable disease management

**DOI:** 10.3389/fpls.2026.1924185

**Published:** 2026-09-08

**Authors:** Shahad Alqahtani

**Affiliations:** College of Science and Humanities, Department of Biology, Shaqra University, Dawadmi, Saudi Arabia

**Keywords:** anti-virulence, biofilms, nano-biopesticides, nanotechnology, plant pathogenic bacteria, Qs, quorum quenching, sustainable agriculture

## Abstract

Bacterial diseases are a major cause of crop losses worldwide, threatening food security and the sustainability of agricultural production. Traditional management practices heavily rely on antibiotics, resistant strains and copper-based compounds but their continued efficiency is hampered by antimicrobial resistance, ecological issues and rapid evolution of pathogens. Nano-enabled anti-virulence strategies offer a novel approach that specifically targets bacterial virulence instead of the killing of the pathogens alone, which can potentially reduce the pressure for resistance development. This review rigorously investigates mechanistic basis of nano-facilitated interference in bacterial communication and pathogenicity in the plant pathosystems. We evaluated the impact of metallic, metal oxide, silica-based, carbon-based and polymeric nanomaterials on quorum sensing (QS), biofilm formation, extracellular polysaccharide synthesis, motility, secretion pathways, phytotoxin production and host colonization. Particular emphasis is placed on nanoparticle–pathogen interactions, nano-enabled delivery of quorum quenchers, RNA molecules and biological agents, microbiome compatibility, and the integration of these processes within a mechanistic anti-virulence framework. Current evidence indicates that nanomaterial efficacy is strongly influenced by physicochemical properties, target virulence mechanisms, delivery efficiency, and environmental conditions; therefore, no single nanoplatform is universally optimal across plant–pathogen systems. Although smart delivery systems provide opportunities to simultaneously interfere with interconnected virulence pathways, their practical application remains constrained by field stability, biosafety, non-target effects, scalability, and regulatory uncertainty. Overall, advancing nano-enabled anti-virulence technologies from laboratory efficacy toward sustainable crop protection will require mechanism-guided nanomaterial design, microbiome-compatible formulations, rigorous field validation, and comprehensive environmental risk assessment.

## 1 Introduction

Plant bacterial diseases remain major biological constraints to global agricultural productivity resulting in significant yield losses and quality deterioration of crops in different agroecosystems (Song et al., 2025b; Xiong et al., 2026). Bacterial plant diseases are characterized by high genetic plasticity, fast evolution potential and complex host colonization mechanisms, allowing them to adapt to changing environmental conditions and escape from classical disease control strategies (Arif, 2024a; Cucu et al., 2025). Major bacterial pathogens infect economically important crops like cereals, vegetables, fruit trees, ornamentals and industrial crops. The pathogens include *Xanthomonas* spp., *Pseudomonas syringae*, *Ralstonia solanacearum*, *Pectobacterium* spp., *Dickeya* spp. and *Agrobacterium* spp (De Pessemier et al., 2026). These pathogens cause destructive diseases such as bacterial wilt, bacterial spot, soft rot, black rot, crown gall, and canker throughout the world.

Bacterial pathogens employ complex communication networks to coordinate behaviors involved in colonization, survival and pathogenicity. These communication systems regulate key processes including biofilm formation, extracellular polysaccharide production, motility, activation of secretion systems, toxin biosynthesis and stress adaptation (Papaneophytou, 2026). Hence, an alternative paradigm for disease management has emerged, namely targeting bacterial virulence mechanisms rather than killing bacterial cells directly (Yarahmadi et al., 2025). Recently, much attention has been focused on the disruption of bacterial communication systems, in particular, QS, a master regulator of many virulence-associated functions. Inhibition of QS can effectively reduce the pathogenicity without necessarily inhibiting the bacterial growth by disrupting the signal production, signal transmission or signal perception. Unlike conventional bactericides that generally kill bacteria, QS inhibition disrupts bacterial communication networks, which reduces virulence and maintains beneficial bacteria and also decreases the evolutionary pressure for development of resistance (Wu et al., 2026).

Nanomaterials have increasingly been shown to interfere with QS signaling, inhibit biofilm formation, disrupt extracellular polymeric matrices, reduce bacterial motility, suppress secretion systems, and modulate the expression of virulence-associated genes. Importantly, these virulence determinants are functionally interconnected through complex regulatory pathways, such that disruption of one process frequently affects several downstream pathogenic traits. Such systems-level interference sets nano-enabled anti-virulence strategies apart from conventional antibacterial strategies that mainly target bacterial survival instead of coordinated virulence regulation (Vadakkan, 2026). In addition, nanotechnology allows the creation of sophisticated delivery systems that can deliver quorum-quenching enzymes, antimicrobial peptides (AMPs), RNA molecules and gene editing tools directly to target pathogens. These nano-enabled approaches offer opportunities for precise and controlled manipulation of bacterial behavior, while reducing chemical inputs and improving environmental sustainability (Malook et al., 2026). Nanomaterials are involved in the control of bacterial infections by reducing pathogen virulence, modulating plant-associated microbial populations and enhancing host defense mechanisms (Zhang et al., 2025). It is important to understand the complex relationships between nanotechnology and ecology and agricultural sciences for developing safe and efficient disease management systems that conserve beneficial microbes and target bacterial pathogens.

Although substantial progress has been made, important knowledge gaps remain concerning the mechanisms by which nanomaterials disrupt bacterial communication networks and virulence pathways (Azeem et al., 2025). This review article covers the recent advances in nano-enabled anti-virulence approaches for the management of bacterial diseases in plants. It critically discusses the mechanisms of interference of nanomaterials with QS and other virulence-associated pathways, the molecular basis of bacterial communication and regulation of virulence, and emerging nano-delivery platforms for anti-virulence molecules. Finally, the review highlights the major scientific, environmental, regulatory and translational challenges that need to be addressed for the practical application of these technologies in sustainable plant disease management.

## 2 Bacterial virulence and communication systems in plant pathosystems

Plant pathogenic bacteria can tightly regulate the expression of different virulence factors through complex regulatory networks to sense host signals, invade and colonize host tissues, and escape plant defense responses defense. Bacterial phytopathogens rely on well-organized regulatory systems that integrate environmental sensing and cell-to-cell communication in order to flexibly control virulence, host invasion and adaptation to changing environmental conditions (Saati-Santamaría et al., 2026). Many of these collective actions are mediated by QS-based signaling mechanisms controlling the expression of important virulence factors such as biofilm formation, extracellular enzyme production and phytotoxin synthesis. Knowledge of the regulatory mechanisms employed by many bacterial pathogens that utilize conserved infection strategies across a range of crop species is critical for the development of novel nano-enabled strategies to disrupt bacterial signaling, reduce virulence and alleviate plant diseases (Zhang et al., 2024b).

**Figure 1:**
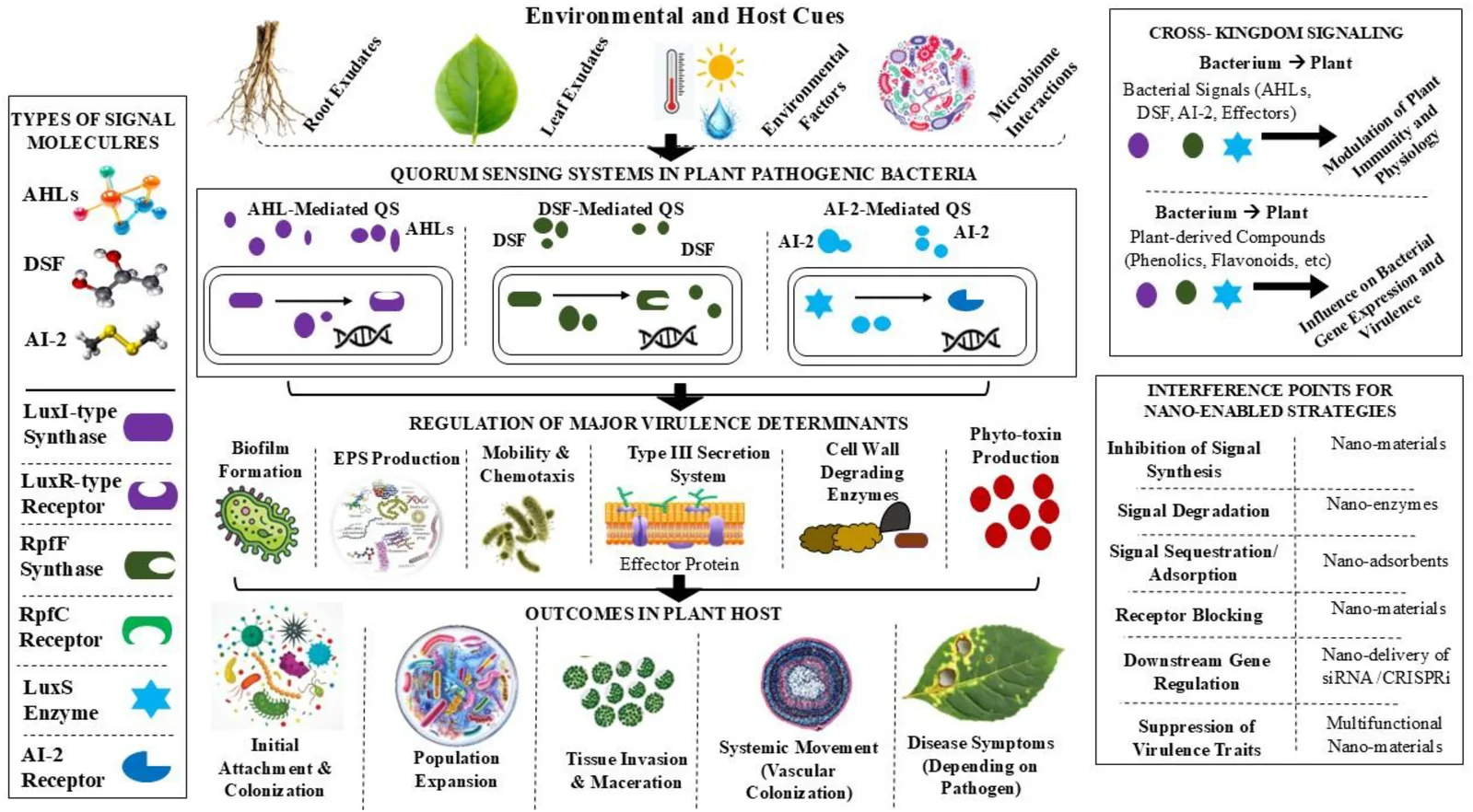
Overview of the concepts of bacterial communication and virulence networks in plant pathosystems. AHL, DSF and AI-2 have been defined and characterized. The QS systems integrate environmental and host-derived cues to regulate key virulence factors including biofilm formation, extracellular polysaccharide production, motility, secretion systems, degradative enzymes and toxin biosynthesis ultimately driving host colonization and disease progression.

Plant pathogenic bacteria are a taxonomically diverse group of microbes that have the potential to infect almost all cultivated crop species (Gao et al., 2026). These conserved pathogenic characteristics are summarized in **Table 1**; **Figure 1** and provide a conceptual basis for understanding the simultaneous targeting of diverse bacterial pathogens via shared regulatory pathways by nano-enabled anti-virulence strategies.

**Table 1:** Major plant pathogenic bacteria, their virulence determinants, communication systems, and potential nano-enabled intervention targets.

Plant pathogen	Major diseases	Key virulence factors	Communication system	Role in pathogenicity	Potential nano-enabled anti-virulence targets	References
*Xanthomonas* spp.	bacterial blight, bacterial spot, black rot, citrus canker	xanthan gum (EPS), biofilms, Type III secretion system (T3SS), extracellular enzymes	Diffusible signal factor (DSF)	regulates biofilm formation, EPS production, host colonization, and virulence gene expression	DSF disruption, EPS inhibition, biofilm destabilization, T3SS suppression	(Ryan et al., 2011; Soares et al., 2026)
*Pseudomonas syringae*	leaf spots, cankers, blights	phytotoxins (coronatine, syringomycin), T3SS effectors, biofilms	acyl homoserine lactones (AHL)-mediated QS and regulatory networks	facilitates immune suppression, toxin production, epiphytic survival, and tissue colonization	QS inhibition, toxin suppression, biofilm disruption, effector delivery interference	(Xin et al., 2018; Sun et al., 2025)
*Ralstonia solanacearum*	bacterial wilt	EPS, biofilms, motility, T3SS, cell wall-degrading enzymes	Phc QS system	controls xylem colonization, vascular blockage, motility, and virulence expression	EPS reduction, biofilm inhibition, QS interference, motility suppression	(Hussain et al., 2021; Chachar et al., 2025)
*Pectobacterium* spp.	SOFT rot, blackleg	pectinases, cellulases, proteases, biofilms	AHL-mediated QS	coordinates production of degradative enzymes and tissue maceration	AHL degradation, enzyme suppression, biofilm disruption	(Anandan and Vittal, 2025)
*Dickeya* spp.	soft rot, stem rot	cell wall-degrading enzymes, motility, biofilms	AHL-mediated QS	Regulates enzyme secretion, biofilm formation, and host tissue invasion	Quorum quenching, enzyme inhibition, motility reduction	(Zhang et al., 2021; Liu et al., 2022)
*Agrobacterium tumefaciens*	crown gall	Ti plasmid transfer system, biofilms, virulence (vir) genes	AHL-mediated QS (TraI/TraR)	Controls plasmid conjugation, tumor formation, and host colonization	QS disruption, plasmid transfer inhibition, biofilm suppression	(Subramoni et al., 2014; Barton et al., 2021)

### 2.1 Major virulence factors in plant pathogenic bacteria

Virulence factors are specialized bacterial traits that increase the pathogen’s ability to infect plants, overcome host defense, acquire nutrients, and spread within host tissues. Virulence factors enable bacterial infection and nutrient acquisition, and regulatory factors are key targets for anti-virulence strategies (Abdullah et al., 2017). These factors rarely act in isolation but rather in concert with interconnected regulatory networks that serve to maximize pathogenic success.

#### 2.1.1 Biofilm formation

Biofilms consist of ordered microbial populations embedded in self-produced extracellular matrices of polysaccharides, proteins, lipids, and extracellular DNA. Formation of biofilms is one of the most important virulence strategies of plant-pathogenic bacteria. Biofilms provide a protective matrix that enables stable attachment, enhanced resistance to environmental and host stresses, improved acquisition of nutrients and persistent colonization of plant tissues (Flemming et al., 2023; Qureshi et al., 2026). Biofilms have been shown to be involved in disease development in vascular pathogens such as *Ralstonia solanacearum* and *Xylella fastidiosa* by blocking the xylem (Ramesh et al., 2026). Biofilm formation is highly regulated by QS systems, making it a particularly attractive target for nano-enabled anti-virulence technologies.

#### 2.1.2 Extracellular polysaccharides

EPS are carbohydrate polymers of high molecular weight, which are secreted by bacterial cells into the environment. EPS are the structural matrix of bacterial biofilms and are responsible for attachment to surfaces, water retention, nutrient sequestration and protection from environmental insults and host defense. Among the different components of the EPS matrix, there are exopolysaccharides such as the xanthan gum produced by the Xanthomonas spp. The EPS of Ralstonia solanacearum is an important virulence factor that contributes to host colonization, vascular occlusion and protection from host immune responses and environmental stresses (Flemming et al., 2025).

#### 2.1.3 Cell wall-degrading enzymes

The first physical barrier encountered by invading pathogens is the plant cell wall. Many bacterial pathogens secrete copious amounts of degradative enzymes that can break down structural plant polymers, thus overcoming this barrier. The principal enzymes involved in the breakdown of the main cell wall are pectinases, cellulases, polygalacturonases, xylanases and proteases (DeTemple and Zabotina, 2025). These enzymes are of particular interest in soft rot pathogens such as *Pectobacterium* and *Dickeya*, where production is often regulated by QS.

#### 2.1.4 Phytotoxins

Representative phytotoxins produced by plant pathogenic bacteria and their biological effects are summarized in **Table 2**.

**Table 2:** Phytotoxins produced by bacteria and their biological impression on plants.

Pathogen	Toxin	Biological effect	References
*Pseudomonas syringae*	*Coronatine*	stomatal reopening and suppression of plant immunity	(Kemppinen et al., 2025)
*Pseudomonas syringae* pv. *phaseolicola*	*Phaseolotoxin*	inhibition of arginine biosynthesis	(Nielipinski et al., 2023)
*Pseudomonas syringae*	*Syringomycin*	membrane disruption	(Grenz et al., 2025)
*Burkholderia glumae*	*Toxoflavin*	oxidative damage	(Takita et al., 2025)

#### 2.1.5 Type III secretion system

The T3SS is one of the most complex virulence strategies employed by Gram-negative plant pathogenic bacteria. The needle-like device works like a molecular syringe, allowing bacteria to inject effector proteins directly into host cells (Asif et al., 2025). T3SS effectors of major bacterial pathogens including *Xanthomonas* spp., *Pseudomonas syringae*, *Ralstonia solanacearum* and *Erwinia amylovora* suppress host immune responses and promote pathogen establishment (Niroula et al., 2026). Although the T3SS is broadly conserved, its regulation is species-specific and is controlled by distinct regulatory networks that integrate environmental signals, quorum sensing and host-derived cues to optimize effector secretion during infection. Since T3SS functionality is often critical for successful infection, inhibition of secretion systems has become a viable anti-virulence strategy.

#### 2.1.6 Motility and chemotaxis

Motility allows bacterial pathogens to find sites of infection, move along plant surfaces, and colonize host tissues. Swimming allows movement to host-derived chemotactic signals, swarming promotes rapid surface colonization and synchronized population increase, and twitching allows firm adhesion and localized movement within plant tissues. Root exudates act as important chemotactic cues for various soilborne bacterial pathogens (Riseh et al., 2025). Many studies have demonstrated that interference with these motility processes significantly reduces bacterial virulence, making them attractive targets for nanotechnology-based anti-virulence strategies.

### 2.2 QS: The communication language of plant pathogens

QS is a cell-to-cell communication system used by bacterial pathogens to coordinate group behavior in response to population density by producing and detecting signaling molecules called autoinducers. These molecules accumulate as bacteria grow and upon reaching a critical concentration activate transcriptional regulators that change gene expression (Raya et al., 2025; Xu et al., 2026). This synchronization allows for expression of virulence factors by pathogens when the pathogen is present in sufficient numbers to overcome host defense.

#### 2.2.1 N-Acyl homoserine lactones

Among the Gram-negative plant pathogenic bacteria, the AHL-mediated QS system is the most well-studied system of communication. AHL molecules contain a conserved homoserine lactone ring attached to acyl side chains of different lengths and structures (Urbauer et al., 2026). These molecules can diffuse freely through bacterial membranes and will build up in proportion to population density. AHL systems regulate biofilm formation, motility, EPS production, enzyme secretion and virulence gene expression. AHL-mediated signaling is used by many plant pathogens including species of *Pectobacterium, Dickeya, Agrobacterium* and *Pseudomonas* (Anandan and Vittal, 2025; Singh and Singh, 2025).

#### 2.2.2 Diffusible signal factor

The DSF system is common among the *Xanthomonas* species and consists of molecules derived from unsaturated fatty acids. It controls similar physiological processes, notably in *Xanthomonas campestris* and is essential for disease development (Wijesinghe et al., 2025).

#### 2.2.3 Autoinducer-2

AI-2 is a universal signal modulating community coordination and mixed-species biofilm development in diverse microbial communities through interspecies communication (Guo et al., 2026; Xu et al., 2026).

#### 2.2.4 Cross-kingdom signaling in plant–microbe interactions

Recently, communication has been found not only between bacteria but also among plants and beneficial microorganisms, leading to complex cross-kingdom signaling networks. Compounds from plants can copy or interfere with bacterial signals, and bacterial signals can influence plant physiology (Arif et al., 2021; Song et al., 2025a; Zhou et al., 2026). This dynamic interplay offers opportunities for targeted disruption with nanotechnology and enhances our understanding of plant-pathogen interactions.

Bacterial pathogenicity involves biofilm development, extracellular polysaccharide production, motility, secretion systems, and phytotoxin biosynthesis, but nano-enabled intervention targets them differently. Biofilm formation and quorum sensing appear to be the best targets for intervention as they affect multiple downstream virulence traits. Disruption of bacterial communication can have broader anti-virulence effects than blocking specific factors. Blocking single determinants such as phytotoxin production or cell wall-degrading enzymes may decrease disease severity but not pathogen colonization, since other virulence pathways are still active. Such hierarchical organization of bacterial virulence implies that nanoparticles targeting upstream regulatory networks will be more effective and longer lasting in inhibiting disease than those targeting downstream processes. Hence, multifunctional nanoplatforms targeting quorum sensing, biofilm formation and secretion systems with minimal bactericidal pressure and preservation of beneficial microbiota should be developed in future research. Collectively these observations indicate that biofilm formation, EPS production and secretion systems should not be considered as independent virulence determinants but rather as parts of interconnected regulatory networks. Therefore it is anticipated that disruption of the upstream signaling pathways would result in a greater anti-virulence activity than inhibition of individual downstream virulence traits (Letizia et al., 2025).

## 3 Nanotechnology platforms for plant disease management

The continuous progress of nanotechnology has facilitated the creation of new platforms for the control of plant diseases, which target bacterial virulence, communication, and host–pathogen interactions, beyond classical antibacterial approaches. Nanomaterials are unusual because of their tiny size and large surface area; they may interact with pathogens and plants at a molecular level which is useful in targeted control of diseases (Farrukh et al., 2025). Nanomaterials of many origins including metallic nanoparticles, carbon-based materials and hybrid composites have been shown to be effective against bacterial growth and biofilm formation as well as to increase plant defense responses showing their promise in agriculture (Sodhi et al., 2025; Salam et al., 2026). The nanomaterials used against plant pathogenic bacteria and their functional properties are given in **Table 3**. The biological behavior of nanomaterials is determined by their physicochemical properties and not by their chemical composition. Size, shape, surface charge, crystallinity and catalytic activity of particles can strongly affect interactions with bacteria. Thus, nanoparticles of the same material might show different anti-virulence effects because of the differences in synthesis, coating and surface functionalization (Vu Thanh et al., 2025). However, despite their potential anti-virulence properties, metallic nanoparticles may have deleterious effects on non-target microorganisms and plant physiological processes under certain environmental conditions, owing to their life span, ion discharge and generation of reactive oxygen species. Therefore, optimization of dosage, formulation and frequency of application of nanoparticles is important to improve efficacy and decrease environmental hazards (Vadakkan, 2026).

**Table 3:** Nanomaterials used for managing plant pathogenic bacteria and their functional properties.

Nanomaterial category	Representative nanomaterials	Major functional properties	Anti-virulence targets	Advantages	Limitations	References
Metallic NPs	silver (AgNPs)	ROS generation, membrane disruption	biofilms, QS, EPS, motility	broad-spectrum activity	potential phytotoxicity at high doses	(Rodrigues et al., 2024; Sedighi et al., 2024; Khalifa et al., 2025)
Metallic NPs	copper (CuNPs)	enzyme inhibition, oxidative stress	biofilms, QS pathways	improved efficacy over bulk copper	environmental accumulation concerns	(Afrasiabi and Partoazar, 2024; Kalu et al., 2026)
Metallic NPs	zinc oxide (ZnO-NPs)	photocatalytic activity, ROS production	biofilms, communication pathways	low cost, micronutrient benefit	activity influenced by environmental conditions	(Hu et al., 2024; Rajkhowa et al., 2024)
Metallic NPs	titanium dioxide (TiO_2_-NPs)	photocatalytic oxidation	signal molecules, biofilms	high stability	light-dependent activity	(Mehta and Bhushan, 2024; Sellami et al., 2025)
Carbon-based nanomaterials	graphene oxide	signal adsorption, membrane damage	QS molecules, biofilms	large surface area	ecotoxicological uncertainty	(Braylé et al., 2022; McCourt et al., 2022)
Carbon-based nanomaterials	carbon nanotubes	carrier function, membrane interaction	virulence factors, signaling pathways	high loading capacity	production cost	(Ayanda et al., 2024; Afrasiabi et al., 2025)
Carbon-based nanomaterials	carbon quantum dots	fluorescence, antimicrobial activity	communication systems	biosensing potential	limited field studies	(Alavi et al., 2021; Romulo et al., 2025)
Polymeric NPs	chitosan NPs	membrane destabilization, ISR induction	biofilms, virulence genes	biodegradable and biocompatible	lower persistence	(Saleh and Hassan, 2024)
Polymeric NPs	alginate/PLGA NPs	controlled release	delivery of anti-virulence molecules	targeted delivery	formulation complexity	(Sedighi et al., 2024)
Nanoemulsions	essential oil nanoemulsions	sustained release	QS inhibition, biofilm disruption	improved stability of bioactive	surfactant dependence	(Hashem et al., 2025; Almeida et al., 2026)
Lipid-based systems	liposomes, SLNs	encapsulation and targeted delivery	signaling pathways	high biocompatibility	storage stability issues	(Ranjbar et al., 2023; Rehman et al., 2024)
Hybrid nanomaterials	metal–polymer composites	multifunctional activity	multiple virulence determinants	synergistic effects	regulatory challenges	(Adu et al., 2022)
Smart nanoplatforms	stimuli-responsive NPs	triggered release	pathogen-specific targets	precision management	early-stage development	(Nguyen et al., 2025; Ramburrun et al., 2025)

Biodegradable polymeric nanocarriers may be useful in minimizing long-term accumulation in the environment, but detailed studies of degradation products and extensive field trials are needed to confirm long-term biosafety (Sedighi et al., 2024).

## 4 Nano-enabled disruption of bacterial communication systems

**Figure 2:**
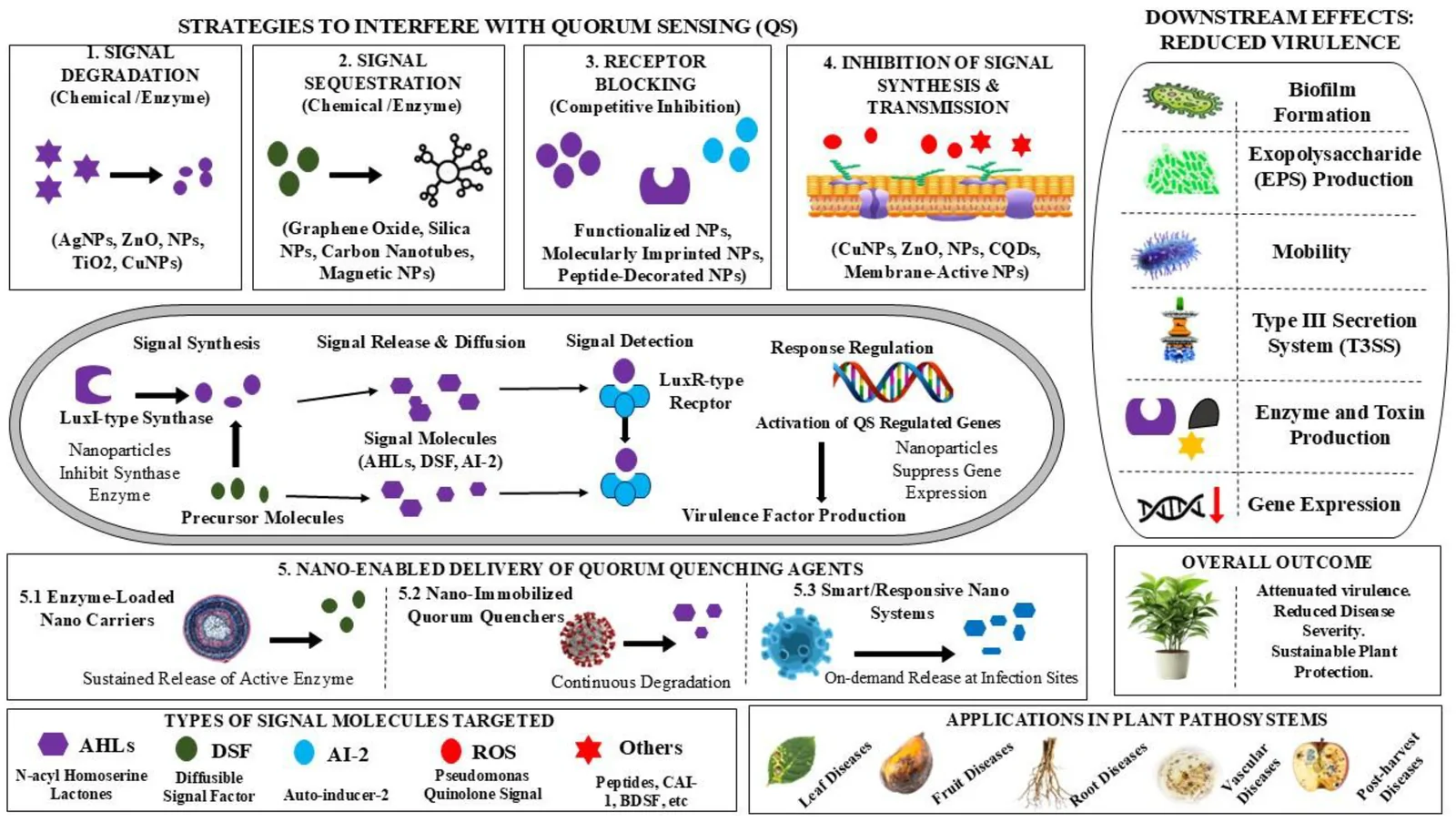
Schematic illustration of nano-enabled disruption of bacterial communication systems in plant pathogenic bacteria. NPs interfere with QS by degrading signaling molecules, adsorbing communication signals, blocking receptor activation and suppressing QS-regulated genes. Collectively, these disruptions reduce biofilm formation, extracellular polysaccharide production, motility, secretion systems activity, and overall bacterial virulence, which will in due course contribute to sustainable disease management.

Development of bacterial diseases of plants relies on establishment of the pathogen and QS for coordination of behaviors such as biofilm formation. Nanotechnology advances provide ways to interfere with QS without damaging bacteria and lowering virulence (Ijaz et al., 2021; Jacobowski et al., 2025). Nano-enabled materials can interact with signaling molecules and thus are promising tools for sustainable management of plant diseases as shown in **Figure 2**; **Table 4**.

**Table 4:** Nano-enabled strategies for disrupting bacterial communication systems in plant pathogens.

Strategy	Nanomaterial examples	Primary target	Mechanism of action	Expected outcome	References
Signal degradation	AgNPs, ZnO-NPs, TiO_2_-NPs	AHLs, DSF, AI-2	ROS-mediated oxidation	reduced signal availability	(Zhang et al., 2024a; Skurková et al., 2025)
Signal sequestration	graphene oxide, silica NPs	QS molecules	adsorption and immobilization	communication blockage	(El-Shetehy et al., 2021)
Competitive inhibition	functionalized NPs	QS receptors	signal mimicry and receptor competition	reduced receptor activation	(Chen et al., 2023; Jiang et al., 2024)
Gene suppression	metallic NPs	QS regulatory genes	downregulation of gene expression	reduced virulence	(Elshaer and Shaaban, 2021; Afrasiabi and Partoazar, 2024)
Signal transmission disruption	membrane-active NPs	signal transport systems	altered membrane permeability	impaired communication	(Eid et al., 2026)
Enzyme-loaded nanocarriers	chitosan, PLGA NPs	signal molecules	controlled enzyme delivery	enhanced quorum quenching	(Zhao et al., 2025)
Nano-immobilized quorum quenchers	hybrid nanocomposites	QS pathways	surface-bound inhibitors	long-term signal disruption	(Sikdar and Elias, 2020)
Controlled-release systems	smart polymeric NPs	multiple communication targets	stimuli-responsive release	sustained anti-virulence activity	(Chen et al., 2023)
Multifunctional nanoplatforms	Hybrid NPs	multiple virulence pathways	simultaneous multi-target disruption	broad-spectrum disease suppression	(Feng et al., 2024)

### 4.1 Nanoparticle-mediated QS inhibition

The NP-mediated QS inhibition is a novel approach to target the bacterial communication systems that regulate virulence rather than to target the bacterial viability (Nithyanand et al., 2025). The aim of this approach is to drive pathogen populations to less virulent strains, with the prospect of reducing disease severity and its ecological impact (Sengupta et al., 2025). QS is inhibited by nanoparticles through several mechanisms, including degradation of signaling molecules, sequestration of signals, and competitive inhibition with receptors.

#### 4.1.1 Signal molecule degradation

One of the simplest strategies to disrupt QS is degradation or chemical modification of signaling molecules before they reach the threshold concentrations necessary to activate virulence genes. Such evidence indicates that ROS-induced signal disruption is a common upstream mechanism for the simultaneous attenuation of multiple QS-regulated virulence traits by metallic NPs (Masood et al., 2024). Degrading signaling molecules is an effective way to prevent bacterial populations from accurately sensing their cell density and to disrupt coordinated virulence responses (Mukherjee and Bassler, 2019). Signal degradation is particularly appealing since it happens extracellularly and doesn’t need to penetrate directly into bacterial cells. Thus, resistant mutants are less likely to be selected for and it is still effective against many species of bacteria.

### 4.2 Suppression of QS-regulated genes

The goal of QS inhibition is to turn off the production of virulence genes. Recent studies have identified several related processes of how NPs impact bacterial gene expression, modulating bacterial virulence, rather than just inhibiting bacterial growth (Lahiri et al., 2021; Juszczuk-Kubiak, 2024). Exposure to these NPs alters gene transcription of biofilm formation, toxin production and motility. Most importantly, metallic NPs have been shown to downregulate key QS genes, which affect virulence and pathogen fitness during colonization of the host (Kaur et al., 2026). These results suggest that NPs could affect intracellular mechanisms, offering a possible strategy to reduce virulence without having to kill bacteria, essentially disarming rather than killing pathogens.

### 4.3 Nanoparticle effects on signal production and transmission

Successful QS in bacteria is not only dependent on the ability to perceive signals but also on the effective production and dissemination of signals. This process may be affected by NPs at different levels. They may alter the integrity of the membrane with consequences on the permeability, the synthesis and the release of signaling molecules. This disruption may affect transport systems, reduce extracellular communication, and change metabolic pathways necessary for signal production (Whiteley et al., 2017; Juszczuk-Kubiak, 2024). Moreover, NPs disrupt biofilms that harbor signaling molecules, leading to a complex disintegration of communication networks rather than simple degradation of signals (Lahiri et al., 2021), which interferes with bacterial communication and coordinated virulence responses.

### 4.4 Emerging nano-quorum quenching technologies

The combination of nanotechnology and quorum-quenching biology has inspired the development of advanced anti-virulence systems that specifically target and disrupt bacterial communication pathways, reducing pathogenicity mediated by quorum sensing (Elmegdar et al., 2024; Juszczuk-Kubiak, 2024). These technologies take advantage of the properties of biological quorum quenchers and the delivery efficiency, stability and targeting capacity of nanomaterials.

#### 4.4.1 Enzyme-loaded nanocarriers

The integration of nanotechnology with quorum-quenching biology has led to the development of advanced anti-virulence technologies that specifically disrupt bacterial communication systems. These systems contain quorum-quenching enzymes (e.g., AHL lactonases and AHL acylases) that degrade signaling molecules to inhibit QS. Free enzymes, however, have problems such as instability and rapid degradation under environmental conditions (Rajkhowa et al., 2024). Quorum-quenching enzymes as a delivery system Polymeric nanoparticles, liposomes and silica nanocarriers are effective delivery systems for quorum-quenching enzymes. They enhance enzyme stability, protect enzymatic activity during transit and allow targeted release at infection sites, improving the inhibition of bacterial virulence in plant pathosystems.

#### 4.4.2 Nano-immobilized quorum quenchers

Another promising option is the immobilization of quorum-quenching agents onto the surface of nanoparticles giving rise to nano-immobilized quorum-quenching agents. Enzymes, peptides, antibodies, or quorum-sensing inhibitors can be immobilized on nanocarriers by physical or chemical means. This immobilization leads to improved stability, bioavailability and controlled release of quorum-quenching agents, enabling the design of multifunctional anti-virulence systems. Compared to their free analogs, these nano-immobilized agents show improved stability, prolonged biological activity, and improved resistance to environmental stresses (Hu et al., 2024; Vadakkan, 2026). Furthermore, the high surface-area-to-volume ratio of nanoparticles allows for the binding of many active molecules, which enhances the anti-virulence efficacy by increasing the local concentration and prolonging the activity.

#### 4.4.3 Controlled release systems

In addition, controlled release nanotechnology is significant for the long-term therapy of the diseases as it releases the active chemicals in response to the environmental changes, thereby reducing the chemical waste and improves the therapeutic efficacy (Beig et al., 2022). It is expected that future developments will emphasize intelligent and stimuli-responsive nanoplatforms that combine quorum-sensing detection with targeted therapeutic delivery for the long-term inhibition of bacterial virulence, better protection of non-target organisms, and accurate disease management in field conditions.

There is no universal nanomaterial that can control all bacterial pathogens. Metallic NPs, especially AgNPs have broad anti-virulence activity via inducing oxidative stress and can successfully inhibit biofilm formation and bacterial motility. However, the use of them may result in phytotoxicity and non-target effects. Targeting quorum sensing and graphene oxide disrupts bacterial communication and biofilm structure. Polymeric nanocarriers such as chitosan and PLGA have low antibacterial activity but are excellent in delivering quorum-quenching enzymes and antimicrobial peptides. Although the mechanisms are described separately, they often occur simultaneously after exposure to nanoparticles. The combination of ROS-mediated oxidation, membrane perturbation, and signal adsorption to inhibit quorum sensing indicates that multifunctional nanoparticles are more likely to achieve durable virulence attenuation than single-mechanism formulations (Tu et al., 2026).

## 5 Nano-enabled suppression of bacterial virulence factors

There is growing evidence that the anti-virulence activity of nanomaterials is dictated not only by their elemental composition but predominantly by their physicochemical properties like particle size, morphology, crystallinity, surface charge, catalytic activity, surface functionalization and colloidal stability. Such properties dictate nanoparticle interactions with bacterial membranes, with EPS, with QS molecules and with intracellular regulatory proteins and thus determine the virulence traits that are preferentially affected. Therefore, the antibacterial activity of nanoparticles with similar properties could be significantly different regarding biofilm formation, motility, secretion systems or bacterial communication. This mechanistic insight underscores that the rational engineering of nanoparticle physicochemical properties, beyond simply the choice of material, is essential for optimizing the efficacy and specificity of anti-virulence action (Elbehiry and Abalkhail, 2025; Singh et al., 2025a).

Among the currently available nanomaterials, metallic nanoparticles usually exhibit the broadest anti-virulence spectrum, as they simultaneously interfere with various cellular processes. AgNPs, CuNPs, ZnO-NPs and TiO_2_-NPs mainly function through mechanisms induced by oxidative stress. Their large specific surface area accelerates the generation of ROS, such as hydroxyl radicals (•OH), superoxide radicals (O_2_•^-^), and hydrogen peroxide (H_2_O_2_), resulting in lipid peroxidation, protein oxidation, enzyme inactivation, DNA damage, and disruption of bacterial redox homeostasis. These sublethal physiological perturbations strongly impair QS-regulated phenotypes prior to complete bacterial death. AgNPs have been shown to inhibit biofilm formation, decrease EPS production, impair swimming and swarming motility and reduce the expression of QS-regulated virulence genes in several gramme-negative bacteria such as *Pseudomonas, Xanthomonas* and *Ralstonia* species in experimental studies (Shah et al., 2019; Awadelkareem et al., 2023). Moreover, ZnO-NPs and TiO_2_-NPs inhibit bacterial communication by ROS-mediated oxidation of extracellular signaling molecules as well as destabilization of bacterial membranes, resulting in attenuation of coordinated virulence responses. Thus, the anti-virulence activity of metallic nanoparticles is not only related to the production of ROS, but also related to their physicochemical properties and the interactions with bacterial regulatory networks (Raghuvanshi et al., 2026).

Carbon-based nanomaterials inhibit bacterial virulence through a fundamentally different mechanism. GO and related nanostructures have highly increased surface areas and large numbers of oxygen-containing functional groups rather than relying on oxidative damage, which enables the adsorption of signaling molecules and direct interactions with bacterial envelopes. Experimental data suggest that GO can bind AHLs, which reduces receptor activation and inhibits QS before the induction of downstream virulence genes. Simultaneously, the sharp edges of graphene nanosheets mechanically rupture bacterial membranes, resulting in weakened surface attachment and inhibited biofilm maturation. These physicochemical interactions are also complementary and explain why GO is often more effective against QS-dependent processes such as bacterial communication and biofilm development than intracellular metabolic pathways (Braylé et al., 2022; Zhang et al., 2022; Papaneophytou, 2026). Overall, these findings indicate that carbon-based nanomaterials act primarily by interfering with bacterial communication networks rather than inducing broad cellular damage, suggesting they are promising candidates for selective anti-virulence strategies with minimal non-target effects. Despite the relatively lower bactericidal activity of the carbon-based nanomaterials, the long-term stability of these materials in the environment and their interactions with the soil microbial population are poorly understood and need to be further investigated in field conditions (Zhang et al., 2022; Elbasuney et al., 2023).

Polymeric nanomaterials represent a major class of nano-mediated anti-virulence strategies. Most biodegradable polymeric carriers, such as PLGA nanoparticles, liposomes and similar polymeric nanocarriers, unlike metallic nanoparticles, cause minimal direct oxidative damage and act as efficient delivery vehicles of anti-virulence agents. They enhance stability, controlled release and localized accumulation of quorum-quenching enzymes, antimicrobial peptides, RNA species and small molecule QS inhibitors. Encapsulation protects these bioactive materials from early deterioration, enhances their biological efficacy in various environmental surroundings and increases their anti-virulence potency. Hence, better inhibition of biofilm formation and quorum sensing-mediated virulence was observed by polymeric delivery systems than their free counterparts, emphasizing the importance of effective delivery of cargo in nano-enhanced anti-virulence strategies. Chitosan nanoparticles have intrinsic antimicrobial and anti-virulence properties apart from being delivery vehicles. The positively charged protonated amino groups of chitosan have electrostatic interactions with the negatively charged bacterial cell envelope, which increases the permeability of the membrane, decreases bacterial adhesion and helps to inhibit biofilm formation and other virulence-associated features. This dual ability makes chitosan-based nanomaterials different from many other polymeric carriers, which mainly act as delivery vehicles (Wypij et al., 2023; Geszke-Moritz and Moritz, 2024).

**Figure 3:**
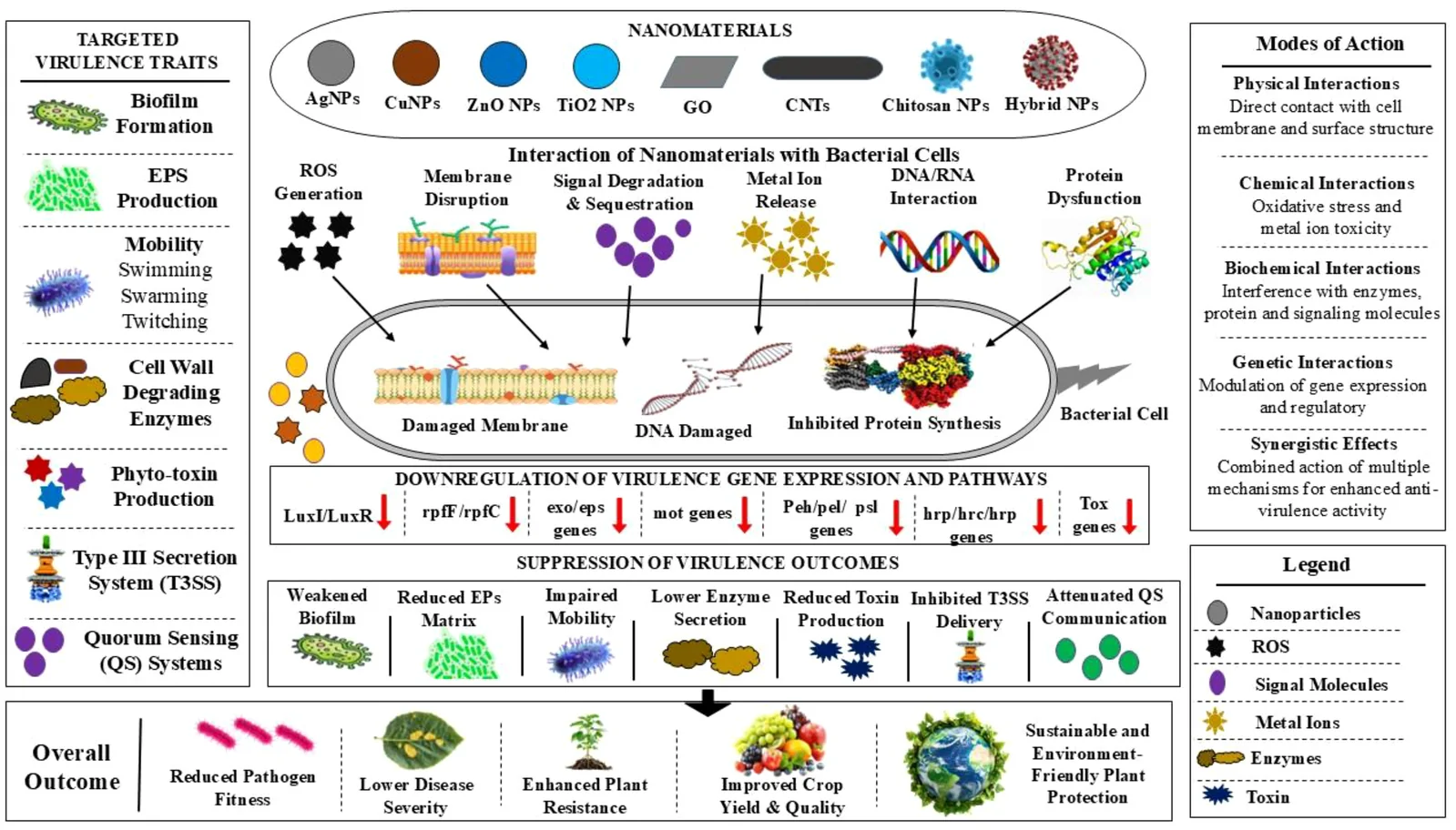
Proposed mechanisms of nano-enabled suppression of bacterial virulence in plant pathosystems. NPs act on bacterial cells by the generation of reactive oxygen species, disruption of the membrane, interference with QS, reduction of EPS and suppression of virulence-associated genes. Together, these effects repress biofilm formation, bacterial motility, production of degradative enzymes and activity of T3SS, ultimately leading to reduced pathogen fitness and disease severity.

Therefore, differences in nanoparticle behavior may be explained by associating their dominant physicochemical qualities with the biological mechanisms responsible for bacterial pathogenicity. Nanoparticles with strong ROS-generating capacity selectively decrease globally controlled virulence determinants, including EPS biosynthesis, phytotoxin generation, motility and secretion systems, as oxidative stress perturbs several regulatory pathways concurrently. On the other hand, nanomaterials with high adsorption capability or changed surface chemistry selectively interfere with extracellular communication by sequestering QS molecules or preventing receptor activation. Carrier-based nanoplatforms provide an extra level of specificity by delivering quorum-quenching enzymes or gene-silencing agents to regions of infection without depending solely on intrinsic antimicrobial action. These molecular distinctions hint that no single nanoparticle is effective against all bacterial pathogens or virulence factors. Recent studies show that different nanomaterials converge on common virulence pathways by different molecular mechanisms. Metallic nanoparticles induce oxidative stress, leading to disturbance of bacterial physiology and carbon-based materials interfere with communication by adsorbing quorum-sensing molecules. Polymeric nanocarriers increase the stability and bioavailability of anti-virulence agents without directly affecting bacterial metabolism. Hybrid nanoplatforms are a combination of these mechanisms to simultaneously disrupt communication, attenuate virulence and deliver therapeutics (Pal et al., 2025; Vadakkan, 2026). This results in a milder disease, less chance of the development of resistance, and promotes a healthier balance of microbes associated with the plant as shown in **Figure 3**.

### 5.1 Inhibition of biofilm formation

Biofilm formation is an important virulence trait that allows bacterial plant pathogens, such as *Xanthomonas* spp., *Pseudomonas syringae*, *Ralstonia solanacearum* and *Agrobacterium tumefaciens*, to establish long-term colonization, adaptation to the environment and quorum-sensing-driven pathogenicity (Wang et al., 2023). Most of the nano-assisted anti-virulence strategies target the early steps of biofilm formation by preventing bacterial attachment, blocking surface attachment and interfering with the production of the extracellular matrix. Metallic nanoparticles like AgNPs, copper CuNPs and ZnO-NPs alter biofilm formation through damaging membrane integrity, inducing oxidative stress and interfering with the synthesis of extracellular polymeric substances At the same time, carbon-based nanomaterials such as graphene oxide and carbon nanotubes inhibit biofilm formation by physical interactions with bacterial cells, adsorption of quorum-sensing molecules and disruption of cell-to-cell communication resulting in decreased bacterial viability, biofilm formation and disease progression. Overall, these results show that nanomaterials prevent the formation of biofilms by several synergistic mechanisms that involve the modulation of bacterial virulence, structural integrity and regulatory networks (Carezzano et al., 2023). Disruption reduces the efficiency of communication between the pathogens and exposes the bacteria to host defense and environmental stresses and is a crucial aspect of nano-enabled anti-virulence strategies as shown in **Table 5**; **Figure 3**.

**Table 5:** Effects of different nanomaterials on bacterial virulence traits in plant pathogens.

Virulence trait	Biological function	Nanomaterials reported	Major mechanism of suppression	Expected outcome	References
Biofilm formation	surface colonization and protection	AgNPs, CuNPs, ZnO-NPs, GO	adhesion inhibition, ROS generation	reduced colonization	(Awadelkareem et al., 2023; Algadi et al., 2024; Qu et al., 2024)
Established biofilms	persistence and stress tolerance	AgNPs, GO, CNTs	matrix degradation	biofilm destabilization	(Sahli et al., 2022; Parvin et al., 2025)
EPS production	biofilm integrity and vascular blockage	AgNPs, ZnO-NPs	gene downregulation	reduced virulence	(Awadelkareem et al., 2022; Abdelgadir et al., 2024)
Swimming motility	pathogen dispersal	AgNPs, CuNPs	flagellar disruption	limited movement	(Pellosi et al., 2025; Suchánková et al., 2026)
Swarming motility	surface colonization	ZnO-NPs, GO	QS interference	reduced spread	(Bhuiyan, 2025; Touati et al., 2025)
Twitching motility	surface exploration and attachment	Metallic NPs	Pilus impairment	reduced biofilm initiation	(Karthikeyan et al., 2025; Scalia and Najmi, 2025)
Cell wall-degrading enzymes	tissue invasion	AgNPs, Chitosan NPs	reduced enzyme synthesis	lower tissue maceration	(Kumar et al., 2022; Sharma et al., 2023)
Phytotoxin production	symptom development	AgNPs, ZnO-NPs	QS suppression	reduced disease severity	(Kalia et al., 2020; Saeki et al., 2022)
T3SS	effector delivery	Metallic NPs, Hybrid NPs	virulence gene downregulation	impaired infection	(Xu et al., 2026)
QS pathways	communication and coordination	GO, AgNPs, Chitosan NPs	signal degradation/sequestration	virulence attenuation	(Liu et al., 2025)

### 5.2 Suppression of extracellular polysaccharide production

EPS are important determinants of pathogenicity, functioning in biofilm formation, surface attachment and protection from host defense. In vascular infections such as *Ralstonia solanacearum*, EPS accumulation leads to wilting symptoms by obstructing water movement in xylem vessels (Chachar et al., 2025). The EPS synthesis is often regulated by QS systems and environmental inputs and the blockage of these pathways can limit the production of polysaccharides. Various nanomaterials, e.g., silver and zinc oxide nanoparticles, showed potential to inhibit EPS formation by down-regulating the expression of the corresponding genes whereas the regulation pathways can be influenced by graphene-based materials. For example, the gum gene cluster (gumB and gumD) involved in xanthan gum biosynthesis in *Xanthomonas* spp. has been silenced, resulting in reduced EPS production and virulence. Similarly, disruption of the phcA-regulated EPS pathway in *Ralstonia solanacearum* results in impaired biofilm formation and vascular colonization (Roy et al., 2023). Lower EPS production interferes with the capacity of bacteria to form biofilms and adhere, making them more vulnerable to plant defense.

### 5.3 Inhibition of bacterial motility

Bacterial motility plays a major role in pathogen dissemination, host localization and tissue invasion. The current evidence suggests that the main mechanism of nanoparticle-induced motility inhibition is the disruption of flagellar assembly and membrane function, while the effect on Type IV pili-mediated twitching motility and cellular energy metabolism is secondary and less well understood (Stabryla et al., 2021; Suchánková et al., 2026). This inhibition of movement is important in controlling the spread of plant diseases.

### 5.4 Suppression of cell wall-degrading enzymes

Bacterial pathogens invade plant tissues by means of cell wall degrading enzymes (CWDEs) which cause tissue maceration and disease symptoms. Plant cell wall components are degraded by enzymes such as pectinases, cellulases, polygalacturonases, and proteases, with pathogens such as *Pectobacterium* spp. and *Dickeya* spp. that rely heavily on these for pathogenicity. Production of CWDEs is generally regulated by quorum-sensing systems that coordinate the expression of enzymes when bacterial populations reach levels that can overcome host defense. Nanomaterials can inhibit these activities by down-regulating the expression of virulence-related genes and perturbing quorum-sensing regulatory mechanisms which in turn lead to reduced transcription and consequent production of CWDEs (Wang et al., 2024). Besides, NPs can induce oxidative stress, which further inhibits enzyme activity and secretion efficiency. Inhibiting these degradative enzymes minimizes tissue destruction and thus limits disease progression in susceptible crops.

### 5.5 Inhibition of phytotoxin biosynthesis

Phytotoxins are secondary metabolites produced during bacterial infections that cause direct damage to plant tissues and interfere with physiological functions. They are involved in symptom development, inhibition of plant immunity and pathogen colonization. Prominent examples are coronatine and phaseolotoxin produced by *Pseudomonas syringae* and toxoflavin produced by *Burkholderia glumae*. Recent studies show that nanomaterials inhibit the production of phytotoxins through interference with the regulatory pathways that control toxin synthesis, rather than through direct bactericidal effects (Chen et al., 2022; Zhang et al., 2023). Nanomaterials can diminish the virulence of bacteria by targeting the regulatory systems involved in the manufacturing of phytotoxins and without compromising the survival of bacteria.

### 5.6 Targeting T3SS

The T3SS is a complex virulence apparatus used by Gram-negative plant pathogens as a molecular syringe to inject effector proteins into host cells. The proteins alter host physiology, dampen immune responses and produce circumstances conducive for bacterial development. T3SS activity is required for efficient infection (Asif et al., 2025; Su et al., 2025). Recent research indicates that NPs can alter T3SS assembly, stability and gene expression, leading to downregulation of regulatory genes and a reduced delivery of effectors and pathogenicity. AgNPs are generally the broadest spectrum anti-virulence nanomaterials available to date, while GO is more specific in its disruption of quorum sensing. In contrast, polymeric nanocarriers have only relatively little intrinsic activity against virulence but significantly improve the delivery efficiency of biological agents (Lahiri et al., 2021; Manisha et al., 2024). Targeting T3SS is a potential strategy for broad-spectrum anti-virulence therapy, and future efforts will focus on creating nanomaterials to selectively target secretion system components and associated regulatory networks.

The comparison underlines that no nanoplatform can optimize efficacy, selectivity, durability, biosafety and scalability simultaneously. Generally, metallic nanoparticles displayed the highest anti-virulence activity, but at the same time, they may also raise concerns regarding the generation of ROS and environmental persistence. Carbon-based and polymeric nanoparticles, on the other hand, give superior selectivity and less non-target impacts. Hybrid and stimuli-responsive systems integrate many processes for increased accuracy and prolonged action. Thus, the choice of an appropriate nanoplatform should be governed by the main virulence strategy of the target pathogen, the intended agricultural use and environmental safety considerations, and not only by antimicrobial potency (Rodrigues et al., 2024).

## 6 Smart nanocarriers for anti-virulence molecule delivery

**Figure 4:**
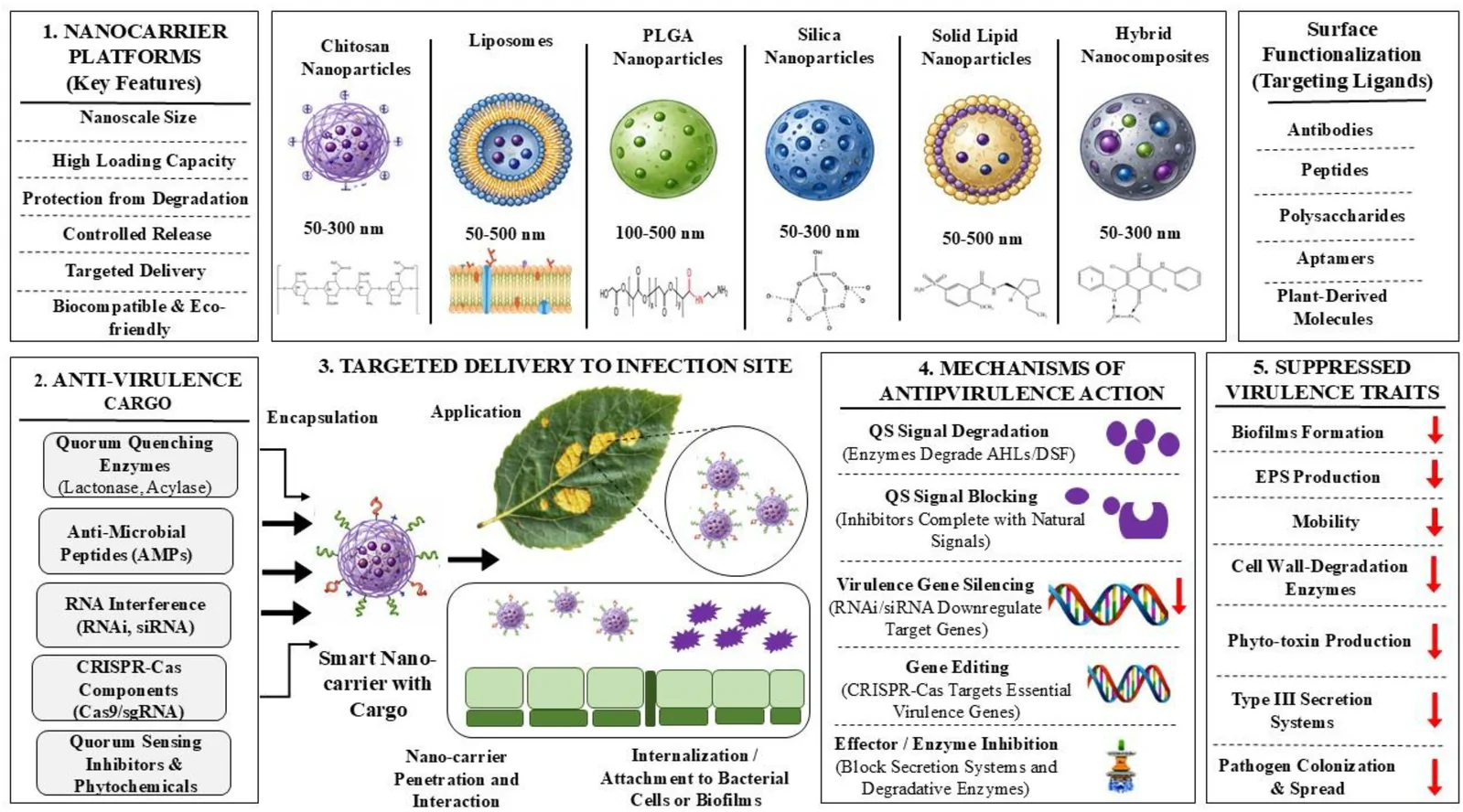
Conceptual framework illustrating smart nanocarrier-based delivery of anti-virulence agents for sustainable management of bacterial plant diseases. Nanocarriers such as chitosan NPs, liposomes, PLGA NPs, silica NPs, and hybrid nanocomposites protect and transport quorum-quenching enzymes, AMPs, RNA molecules, and CRISPR components to infection sites. Targeted delivery disrupts bacterial communication systems, suppresses biofilm formation, silences virulence genes, and inhibits secretion systems, ultimately reducing disease severity and enhancing crop resilience.

The successful implementation of anti-virulence strategies relies on the effective delivery of bioactive compounds to pathogens at the site of infection. Although several anti-virulence agents have been shown to be effective *in vitro*, their application is frequently hindered by poor stability, rapid degradation, low bioavailability and poor target specificity (Filipić et al., 2024). Therefore, intelligent nanocarrier systems have been designed for the protection and controlled release of these substances. Nanomaterial-based platforms loaded with bioactive agents, such as quorum-quenching enzymes and AMPs, improve the stability of treatment, increase delivery efficiency, and mitigate adverse effects on beneficial microbes and the surrounding environments (Zarepour et al., 2024; Jacobowski et al., 2025). Also, recent advances in environmentally responsive delivery methods have opened the way to sustainable crop protection as shown in **Figure 4**; **Table 6**.

**Table 6:** Smart nanocarrier systems for delivery of anti-virulence agents against plant pathogenic bacteria.

Anti-virulence agent	Nanocarrier system	Primary target	Mechanism	Expected outcome	References
AHL lactonase	chitosan NPs	AHL signals	signal degradation	QS suppression	(Boban et al., 2023; Rajkhowa et al., 2024; Patil and Shevate, 2025)
AHL acylase	silica NPs	AHL molecules	signal cleavage	communication disruption	(Naga et al., 2023; Sompiyachoke and Elias, 2024)
AMPs	liposomes	biofilms, membranes	membrane destabilization	reduced virulence	(Makhlouf et al., 2023; Ramôa et al., 2023)
AMPs	PLGA NPs	multiple virulence traits	sustained release	disease suppression	(van Gent et al., 2021; Ramôa et al., 2023)
RNAi molecules	chitosan nanocarriers	virulence genes	gene silencing	reduced pathogenicity	(Mujtaba et al., 2021; Xing et al., 2025)
siRNAs	lipid NPs	QS and EPS genes	mRNA degradation	virulence attenuation	(Tam et al., 2013; Sato et al., 2016)
CRISPR-Cas systems	hybrid nanocarriers	virulence determinants	gene editing	long-term suppression	(Al-Ouqaili et al., 2025; Selim et al., 2025)
QS inhibitors	nanoemulsions	signal receptors	competitive inhibition	reduced communication	(Malešević and Jovčić, 2025)
Multi-cargo systems	smart polymeric NPs	multiple pathways	combined targeting	broad-spectrum control	(Shariati et al., 2022)
Stimuli-responsive cargo	responsive nanocarriers	infection sites	triggered release	precision disease management	(Kalelkar et al., 2022; Wu et al., 2023)

### 6.1 Nanocarrier-based delivery systems

Nanocarriers are nanoscale delivery vehicles that can encapsulate and deliver biologically active compounds to specific targets, thus improving the effectiveness of anti-virulence strategies for plant disease management. They enhance the solubility, bioavailability and stability of encapsulated agents and offer sustained and controlled release (Hussain et al., 2026). Various kinds of NPs like chitosan, silica NPs and liposomes are used to protect active chemicals from external stresses and also to allow controlled release so that the need for repeated treatments is reduced (Wang et al., 2022). In addition, nanocarriers can be functionalized with ligands that recognize pathogens specifically, and thus they can be used to target pathogenic bacteria while having no effect on beneficial microorganisms in the plant environment. However, there are several challenges to field deployment of these advantages. Environmental factors such as ultraviolet irradiation, rainfall, temperature variation, soil chemistry and interactions with exudates from plants can influence the stability, release kinetics and targeting efficiency of nanocarriers under field conditions (Mgadi et al., 2024; Malook et al., 2026). This new technique provides a more effective and more durable treatment.

### 6.2 Delivery of quorum-quenching enzymes

Quorum quenching enzymes are a promising biological tool for disrupting bacterial communication. These enzymes destroy or alter the quorum-sensing molecules before they can activate virulence pathways, halting disease development and enabling bacterial survival (Hetta et al., 2024). However, free enzymes are generally unstable under agricultural circumstances leading to lower activity due to environmental deterioration.

#### 6.2.1 AHLs

AHL lactonases are enzymes that hydrolyze the lactone ring of AHLs, therefore inactivating these signaling molecules. AHL-mediated signaling controls many virulence factors in gram-negative plant pathogens, and the destruction of these signals by lactonases can lead to a drastic reduction of pathogenicity. AHL lactonases encapsulated in polymeric NPs, silica nanocarriers and liposomes can retain their enzymatic activity and stability in the environment. The controlled release of lactonases at infection sites allows constant degradation of signaling molecules, therefore limiting the buildup of QS signals required for the activation of virulence (Patil and Shevate, 2025). Studies have demonstrated that lactonase-loaded NPs are efficient in reducing biofilm formation, EPS production and the expression of virulence genes in a wide range of plant pathogenic bacteria (Touati et al., 2025). However, encapsulation of the enzyme does not guarantee long term stability under field conditions. Thus, further optimization of nanocarrier compositions and long-term field testing are required to ensure the long-term efficacy of quorum-quenching in various agricultural environments.

#### 6.2.2 AHL acylases

AHL acylases inhibit the QS process by the cleavage of the amide bond between the homoserine lactone ring and the acyl side chain, which leads to an irreversible change and deactivation of the AHL molecules (Utari et al., 2017). Nano-encapsulated acylases show higher stability and sustained biological activity than free enzymes. Coupled to smart nanocarriers, acylases represent a strong tool for persistent quorum quenching and long-term disease suppression. Enzyme-loaded nanocarriers combined with responsive release mechanisms can lead to better performance in field situations by releasing the enzymes only when pathogen populations have reached a threshold level. However, the mass production of acylase-encapsulated nanocarriers presents notable difficulties, as the preservation of enzyme activity throughout formulation, the achievement of high encapsulation efficiency, the consistency between batches, and the long-term storage stability require further improvement before commercial application (Saini et al., 2026).

### 6.3 Nano-delivery of AMPs

AMPs are natural products made by a variety of organisms as part of their innate immune responses. Besides their well-established antimicrobial activity, many AMPs show anti-virulence properties by preventing biofilm formation, disrupting quorum-sensing communication and down-regulating the expression of virulence-associated genes. Antimicrobial activity reduces bacterial viability via mechanisms such as membrane disruption. Anti-virulence activity reduces pathogenicity but does not directly affect bacterial viability. However, practical use of AMPs is often limited by rapid degradation, poor environmental stability and short persistence in field conditions. Nanotechnology offers a promising approach to overcome these limitations via encapsulation of peptides and controlled release of encapsulated peptides. Nanoencapsulation of antimicrobial peptides increases their stability, decreases enzymatic degradation, enhances penetration into biofilms, prolongs release and improves delivery efficiency (Fleitas Martínez et al., 2019). Recent research has indicated that AMP-entrapped nanoparticles are able to reduce bacterial virulence via inhibition of biofilm formation, quorum-sensing mechanisms and production of virulence factors, with any antimicrobial effects serving as an additional means of controlling pathogen numbers. In addition, the combination of antimicrobial peptides with metallic nanoparticles may lead to synergistic effects, enhancing anti-virulence and antimicrobial activity and decreasing the amount of active compounds required for effective disease control (Filipić et al., 2024; Gudepu et al., 2026).

### 6.4 Nano-delivery of RNA-based molecules

RNA technologies are now an indispensable tool to target pathogen genes with high specificity and thus a more precise alternative to conventional chemical treatments (Arif et al., 2018). RNA molecules can efficiently silence genes involved in virulence, communication, biofilm formation and host colonization (Banerjee, 2025). However, the vulnerability of these molecules to environmental nucleases requires protective delivery systems, in which nanocarriers are emerging as a promising solution for RNA-based anti-virulence strategies.

#### 6.4.1 RNA interference

RNAi is a mechanism that silences genes through the degradation of specific messenger RNA sequences. The process of RNAi can be used to target QS, biofilm formation, EPS synthesis, toxin production and secretion systems. The use of NPs for RNAi delivery increases RNA stability and allows uptake by bacterial populations. Various nanocarriers like chitosan nanoparticles, layered double hydroxides, silica nanoparticles, lipid nanocarriers have shown great potential (Condinho et al., 2023; Juszczuk-Kubiak, 2024). The RNAi approaches are highly specific; thus, they can be used to control diseases with specificity against pathogens without affecting beneficial microbial communities.

#### 6.4.2 Small interfering RNAs

Small interfering RNAs (siRNAs) are short double-stranded RNA molecules that promote sequence-specific degradation of target messenger RNAs. Nano delivered siRNAs can selectively down-regulate major virulence genes involved in pathogenicity and communication. Encapsulation protects the siRNA from degradation and results in a prolonged release and greater absorption. Although these methods provide a great deal of specificity to the target, their capacity to alter environmental conditions and their potential effects on non-target microbial communities need to be evaluated further before they can be used widely in agriculture (Sargazi et al., 2022). The integration of nanotechnology and siRNA-based gene silencing is one of the most promising approaches for the development of precision plant disease management and next generation crop protection systems.

### 6.5 CRISPR-based virulence gene targeting through nanocarriers

CRISPR technology has revolutionized the field of genetic engineering, and has paved new ways for the management of bacterial plant pathogens by targeting genes involved in virulence, quorum sensing, biofilm development, and toxin production and secretion mechanisms (Yang et al., 2024). But the successful delivery of CRISPR components remains a major obstacle to agricultural applications. Nanocarriers are a promising technology for protecting the CRISPR cargo from environmental degradation and targeted delivery to pathogen populations. Potential targets include quorum-sensing regulators, signal transduction genes, EPS biosynthesis genes, and toxin biosynthetic pathways. Prospective intelligent nanocarriers could be engineered to specifically recognize infections and subsequently release CRISPR payloads triggered by pathogen-derived signals, unifying nanotechnology, synthetic biology and precision agriculture for enhanced disease control. Nevertheless, successful employment of CRISPR-based nanocarrier systems necessitates careful consideration on biosafety and regulatory issues including the potential for accidental off-target genome editing, environmental persistence of gene-editing components, impact on non-target microbes, and establishment of appropriate regulatory frameworks for real-world applications. Robust risk assessments, biosafety evaluations and harmonized regulatory frameworks will be needed to tackle these challenges before they can be widely used in agriculture (Muñoz et al., 2019; Hejabi et al., 2022).

**Figure 5:**
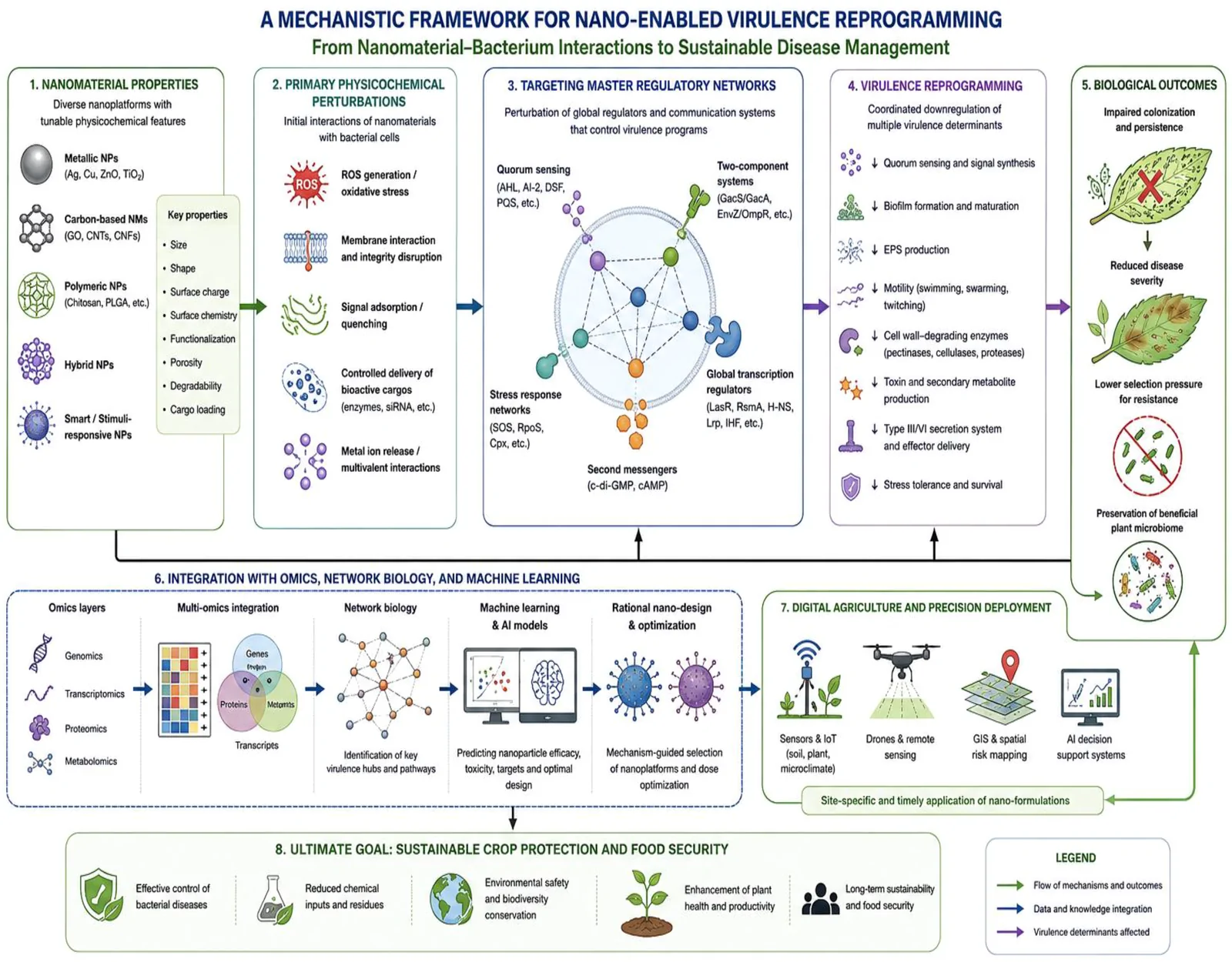
A mechanistic model of nanotechnology-based reprogramming of virulence in plant pathogenic bacteria. Schematic diagram illustrating a proposed concept model showing the modulation of bacterial virulence by different types of nanoplatforms, including metallic NPs, carbon-based NPs, polymeric NPs, hybrid NPs, and stimuli-responsive NPs, through interrelated regulatory pathways rather than direct bactericidal effect. After initial contact of nanomaterials with bacteria, the fundamental physicochemical changes (including modulation of reactive oxygen species, membrane disruption, signal adsorption, regulated cargo delivery and metal ion release) converge on key regulatory networks that control bacterial pathogenicity, including quorum sensing, two-component regulatory systems, stress response pathways, global transcriptional regulators and second messenger signaling. This orchestrated regulatory reprogramming downregulates multiple virulence determinants including biofilm formation, extracellular polymeric substance production, motility, quorum sensing, toxin synthesis, secretion systems and cell wall-degrading enzymes, thereby reducing host colonization, disease severity and the selective force for antimicrobial resistance. The framework highlights the importance of multi-omics, network biology, machine learning and digital agriculture to enable mechanism-driven design of nanoparticles, precision application and large-scale optimization, thus facilitating the translation of the laboratory results to sustainable agriculture and better food security.

Although nano-enabled anti-virulence strategies are often described as having different mechanisms such as ROS generation, quorum sensing inhibition, biofilm disruption, and targeted cargo delivery, these mechanisms are closely related. Based on the current evidence, a mechanistic model is proposed in which various nanoplatforms induce fundamental physicochemical disturbances that are compatible with bacterial regulatory networks, consequently reprogramming several virulence factors and reducing disease progression (Sahu and Satapathy, 2026). This framework integrates existing knowledge into a coherent conceptual model that links nanoparticle properties to sustainable agriculture protection as shown in **Figure 5**.

## 7 Interactions of nanomaterials with plant–pathogen–microbiome systems

The application of nanomaterials for the management of plant diseases is limited to the pathogens but also to the complex ecological interactions that occur in the agroecosystem. Nanomaterials affect not only plant pathogens but also beneficial microorganisms such as plant growth-promoting rhizobacteria, endophytes, mycorrhizal fungi and other members of the plant microbiome that contribute to nutrient cycling, trigger systemic resistance and enable natural disease suppression. Exposure to nanoparticles can thus alter the composition of microbial communities, change the networks of microbial interactions and affect crucial ecological functions, depending on the type, concentration, longevity of nanoparticles and the surrounding environmental conditions. Targeted nanomaterials can selectively kill undesirable bacteria, while beneficial microorganisms remain intact, but nonselective or chronic exposure may reduce microbial diversity, alter functional redundancy, and impact resilience of disease-suppressive soils. Future studies should take an ecological systems approach, including metagenomics, meta transcriptomics, metabolomics and comprehensive field studies to determine effects of nanomaterials on development of microbiomes, ecosystem resilience and sustainable plant health in different agricultural environments (Yadav and Yadav, 2024; Hayat et al., 2026; Raghuvanshi et al., 2026). Progress in sustainable crop protection depends on an understanding of these interactions as shown in **Table 7**.

**Table 7:** Positive and negative effects of nanomaterials on plant-associated microbial communities.

Nanomaterial type	Positive effects on microbiome	Potential negative effects	Beneficial microbial groups affected	Overall implications for plant health	References
Chitosan NPs	stimulate beneficial rhizobacteria, enhance isr	minimal toxicity at recommended doses	*Bacillus*, *Pseudomonas*, *Rhizobium*	improved disease resistance and plant growth	(Chakraborty et al., 2020; Ahmed et al., 2023)
PLGA NPs	controlled delivery with low ecological impact	limited information on long-term effects	PGPR and endophytes	enhanced compatibility with beneficial microbes	(Lee et al., 2022; Oluwole et al., 2025)
Silica NPs	improved nutrient availability and root colonization	potential concentration-dependent stress	Rhizosphere bacterial communities	increased plant vigor and microbial activity	(Meng et al., 2026; Sil et al., 2026)
Silver NPs (AgNPs)	suppress pathogenic bacteria effectively	may reduce microbial diversity at high concentrations	sensitive PGPR populations	strong disease suppression but possible ecological risks	(Afzal et al., 2025; Bhardwaj et al., 2025)
Copper NPs (CuNPs)	reduce pathogen abundance	accumulation may affect beneficial microbes	nitrogen-fixing bacteria	requires careful dosage management	(Ahmad and Ahmad, 2024; Islam, 2025)
Zinc oxide NPs (ZnO-NPs)	promote plant growth and nutrient utilization	oxidative stress in susceptible microbes	Rhizobacteria and endophytes	balanced effects depending on concentration	(Ahmad and Ahmad, 2024; Sodhi et al., 2025)
Titanium dioxide NPs (TiO_2_-NPs)	enhance plant physiological performance	photocatalytic stress on some microbes	mixed microbial communities	potential improvement of crop resilience	(Fathi Qarachal and Alizadeh, 2025; Sodhi et al., 2025)
Graphene oxide	adsorption of pathogen signaling molecules	possible membrane damage to non-target microbes	Rhizosphere bacteria	effective communication disruption but biosafety evaluation needed	(Piperno et al., 2018; Xiao et al., 2019)
Lipid-based nanocarriers	high biocompatibility and targeted delivery	generally low toxicity	beneficial microbial consortia	favorable for sustainable disease management	(Yeh et al., 2020; Ahsan et al., 2024)
Hybrid nanocomposites	multifunctional disease suppression	ecological effects vary by composition	diverse microbial communities	promising but requires comprehensive risk assessment	(Chen et al., 2024; Singh et al., 2025b)
Stimuli-responsive nanomaterials	reduced non-target exposure	limited field validation	broad microbial populations	high potential for precision agriculture	(Ma et al., 2025; Pagano et al., 2025)
Nano-enabled bioformulations	enhanced biological control efficiency	formulation-dependent effects	PGPR, biocontrol bacteria	synergistic disease suppression and plant protection	(Garg et al., 2023; Shafi et al., 2026)

### 7.1 Nanoparticle effects on rhizosphere microbiomes

The rhizosphere is an important component of ecosystems and contains different microbial communities that promote plant health by facilitating nutrient uptake and suppressing disease (Arif, 2025). However, the effect of NPs on these beneficial microorganisms needs to be studied thoroughly. Nanoparticles may induce oxidative stress and modify the nutritional availability; however, the effect is size and concentration dependent. The positive effects of chitosan nanoparticles at optimal concentrations on beneficial microbial populations are seen while the negative effects on microbial diversity and soil fertility are seen when used in higher doses (Ahmed et al., 2023). Therefore, the extensive studies on interactions of nanoparticles with rhizosphere microorganisms under controlled and natural conditions are of utmost importance in the development of ecologically friendly nanomaterials.

### 7.2 Impacts on beneficial plant-associated bacteria

Beneficial microorganisms play an important role in improving agricultural yield, nutrient uptake and plant disease resistance. Important PGPRs include species such as *Bacillus* (e.g., *Bacillus velezensis*, *B. subtilis*, and *B. amyloliquefaciens*), *Pseudomonas* (e.g., *Pseudomonas fluorescens* and *P. putida*), *Azospirillum brasilense, Rhizobium* spp., and *Paraburkholderia* spp. These microbes improve plant health by biologically fixing atmospheric nitrogen, solubilizing phosphate, synthesizing plant hormones, releasing siderophores, inducing systemic resistance and showing antagonistic activity against plant pathogens. Nanoparticles may influence these beneficial bacteria either directly by altering cellular metabolism, membrane stability and gene expression or indirectly by altering the availability of nutrients, root exudation dynamics and microbial competition in the rhizosphere (Das et al., 2025). In contrast, low-toxicity nanomaterials and biodegradable nanomaterials, such as chitosan nanoparticles, could promote microbial colonization and improve beneficial plant–microbe interactions under appropriate conditions (Hussain et al., 2023; Mgadi et al., 2024).

### 7.3 Influence on plant immune responses

Plants have complex immune systems that can sense microbial signals and induce responses that help to prevent infections. NPs can function as elicitors promoting plant immunity via activation of signaling pathways involved in pathogen recognition and stress responses (Joshi, 2025). Exposure to some nanomaterials induced the production of ROS, defense-related enzymes, phenolic compounds and antimicrobial metabolites. Signaling molecules such as salicylic acid (SA), jasmonic acid (JA), ethylene (ET) and ROS regulate defense gene expression and local and systemic coordination of resistance responses (Mukarram et al., 2023).

### 7.4 Nanotechnology and induced systemic resistance

ISR is a defense response of plants that can be induced by beneficial microbes in combination with environmental signals. ISRs do not attack pathogens directly, but rather prime plants to enhance their immune responses. Nanomaterials have been recently shown to induce ISR through the interaction with plants and microbes by activation of key signaling pathways such as ethylene and jasmonic acid. More use of nanotechnology could improve the survival and efficacy of biological control agents, reducing reliance on chemical pesticides and improving sustainable plant resilience (Chen et al., 2025). In the future, new emerging approaches should be developed for the creation of multifunctional nanoplatforms for the delivery of beneficial microorganisms and defense agents.

### 7.5 Balancing disease suppression and microbial ecology

One of the greatest challenges for agricultural nanotechnology is to manage disease without disrupting beneficial bacteria. The balance of microbial variety is directly responsible for soil health, nutrient cycling and disease control, and hence is essential for sustainable crop production. Nanomaterial overuse can disrupt microbial community composition, ecological interactions, and reduce ecosystem resilience. Disease control techniques should thus be disease specific with little impact on beneficial species. These include biodegradable nanomaterials, pathogen-specific targeting systems, controlled release formulations, stimuli-responsive nanoplatforms, microbiome-compatible nanocarriers, and delivery of biological control agents (Arif, 2024b; Iqbal, 2026). Recent breakthroughs in nanotechnology allow the construction of precise delivery systems that are able to identify the signals of the pathogens and release active chemicals at the proper moment (Hajipour et al., 2021), decreasing the exposure to the environment and boosting the compatibility with beneficial microbial populations.

Positive and negative effects of major agricultural nanomaterials on plant-associated microbial communities. The table highlights the importance of balancing pathogen suppression with preservation of beneficial microbiota to ensure sustainable and environmentally responsible nano-enabled disease management.

**Figure 6:**
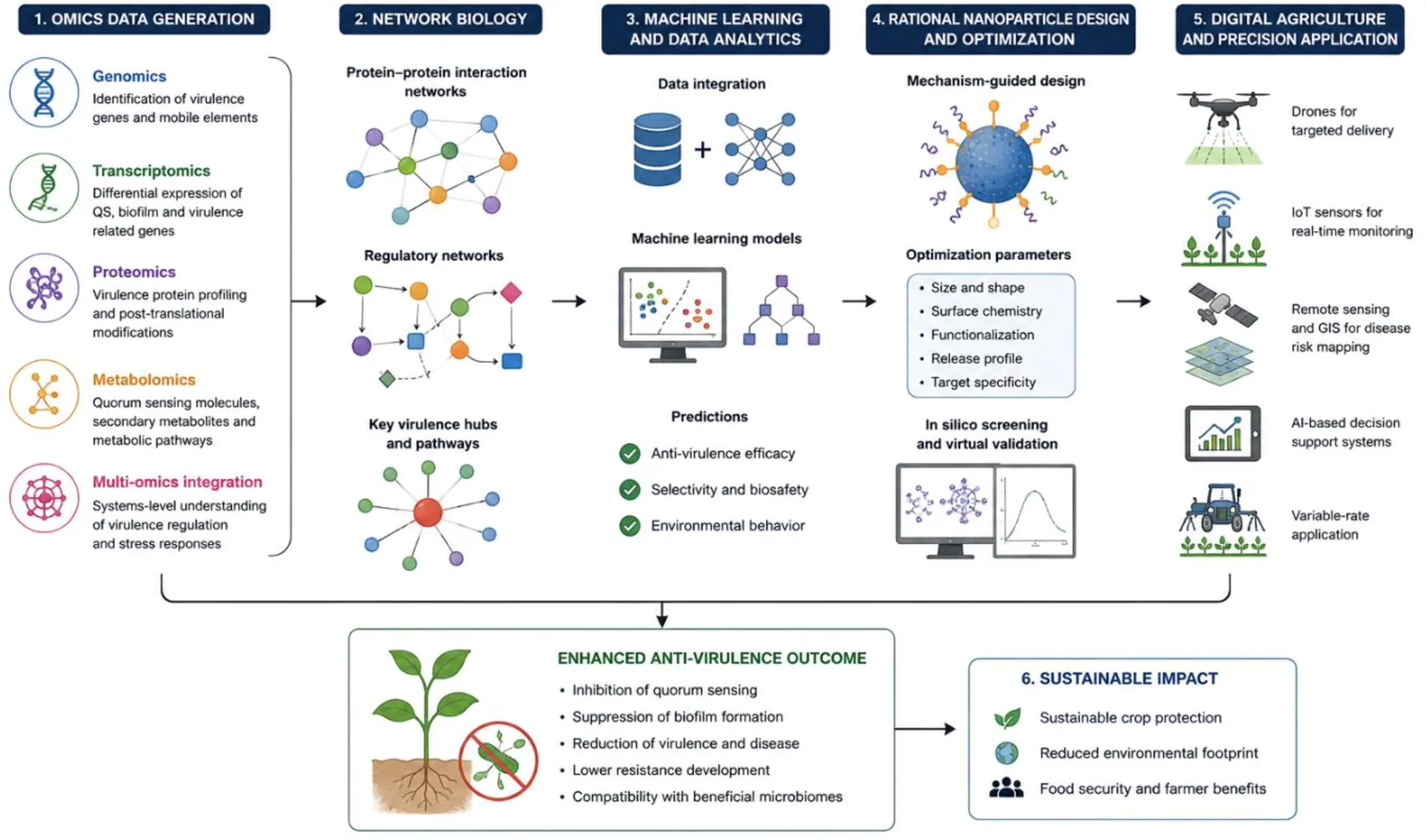
Schematic representation of the identification of bacterial virulence factors and regulatory mechanisms using multi-omics technologies (genomics, transcriptomics, proteomics, metabolomics and integrated multi-omics). These datasets are integrated with protein interaction networks and machine learning techniques for the prediction of essential virulence targets and to support the systematic design of nano-enabled anti-virulence systems with improved physicochemical properties. Engineered nanomaterials are incorporated into digital agriculture systems using drones, IoT sensors, remote sensing technologies, GIS, and AI-based decision-making for precise disease control. The synergy of these allows specific inhibition of quorum sensing, biofilm formation and other virulence traits and enhances crop protection, minimizes ecological impacts, preserves beneficial plant-associated microbiomes and facilitates the translation of laboratory results into sustainable agricultural practices.

Recent advances in systems biology and digital agriculture provide new opportunities to improve the design and application of nano-enabled anti-virulence strategies. Advanced omics technologies, assessment of regulatory networks, machine learning and precision agriculture enable a shift from empirical assessment of nanoparticles to mechanism-informed and data-centric disease management (Abdallah et al., 2026). The combination of these synergistic approaches can improve target identification, optimize nanoparticle design, predict efficacy in different environmental conditions and support large-scale application as shown in **Figure 6**.

In general, nano-enabled anti-virulence strategies have important advantages over conventional bactericidal strategies as they reduce pathogenicity and potentially the development of resistance. However, their successful application in agriculture requires a trade-off between efficacy and biosafety, ecological fitness, microbiome conservation and economic feasibility. Therefore, future studies should evaluate positive and negative effects in real field settings to ensure responsible and sustainable implementation (Fincheira et al., 2023).

## 8 Environmental translation: biosafety, field validation, regulatory challenges, and research gaps

Laboratory experiments have repeatedly demonstrated the potential of nanomaterials to suppress bacterial virulence, but comparatively less evidence exists from greenhouse and field trials. In the field, nanoparticle stability, persistence, mobility, and biological activity may be affected by multiple variables such as changing temperature, UV radiation, rainfall, soil physicochemical properties, microbial community complexity, and plant developmental stage. Therefore, the efficiency achieved in controlled laboratory conditions may not be fully replicated in agricultural systems. Nevertheless, the current greenhouse and field studies mostly support the feasibility of nano-enabled disease management, especially when nanoparticles are incorporated in optimized formulations or targeted delivery systems (Elmer and White, 2018). Therefore, future research needs to focus on multi-location field trials, standard evaluation protocols and long-term evaluation of efficacy, environmental safety and cost-effectiveness to enable commercialization and practical implementation.

They have unique properties that affect mobility and biological interactions unlike conventional pesticides. Such interactions are essential to understand in complex agroecosystems for the evaluation of environmental fate and biosafety of nanomaterials (Salam et al., 2026). The responsible development of the agricultural applications would imply the assessment of the benefits and risks of the nano-enabled strategies to ensure the sustainability of agriculture while safeguarding the health of the ecosystem, eventually leading to safer formulations and informed regulatory frameworks as shown in **Table 8**. Long-term effects are mainly determined by environmental persistence of agricultural nanomaterials. These materials can be present on plant surfaces, penetrate tissues or leach into soil and water systems with their persistence being controlled by factors such as composition, size and environmental conditions (Giese et al., 2018).

**Table 8:** Biosafety concerns and regulatory considerations for agricultural nanomaterials.

Issue category	Potential concern	Environmental or biological impact	Risk mitigation strategy	References
Environmental persistence	long-term nanoparticle stability	accumulation in soil and water	development of biodegradable nanomaterials	(Islam, 2025; Adil et al., 2026)
Soil ecotoxicology	disruption of microbial communities	reduced nutrient cycling and soil health	dose optimization and field monitoring	(Quintarelli et al., 2024; Cao et al., 2025)
Aquatic ecotoxicology	runoff into water systems	effects on aquatic organisms	controlled application and runoff management	(Singh et al., 2023; Zeng et al., 2025)
Non-target microorganisms	toxicity to beneficial microbes	reduced biological control and plant growth promotion	target-specific formulations	(Mgadi et al., 2024; Pagano et al., 2025)
Pollinator exposure	contact with nanoparticle residues	potential physiological stress	pollinator-safe application strategies	(Pagano et al., 2025)
Soil fauna exposure	accumulation in earthworms and invertebrates	altered soil ecosystem functions	comprehensive ecotoxicological testing	(Kapeleka and Mwema, 2024; Doan, 2025)
Food safety	uptake into edible plant tissues	human dietary exposure	residue monitoring and safety assessment	(Abdulraheem et al., 2025; D’Angelo, 2026)
Occupational exposure	inhalation or skin contact during application	human health risks	protective equipment and handling guidelines	(Pietroiusti et al., 2018; Gomez-Villalba et al., 2023)
Regulatory uncertainty	lack of nano-specific legislation	delayed commercialization	harmonized regulatory frameworks	(Kumari et al., 2023; Rasmussen et al., 2025)
Public perception	consumer concerns regarding nanotechnology	reduced market acceptance	transparent communication and education	(Porcari et al., 2019; Joubert et al., 2020)
Economic feasibility	high production costs	limited farmer adoption	cost-effective manufacturing technologies	(Dabare et al., 2025; Khan et al., 2025)
Long-term sustainability	unknown multi-season effects	ecosystem-level consequences	long-term environmental monitoring	(Pagano et al., 2025)

Nanomaterials can affect the cycling of nutrients, respiration of microbes, activity of enzymes and decomposition of organic waste in soil. Soil microorganisms play a crucial role in controlling soil fertility and disease suppressiveness, and changes in microbial community structure can have major ecological consequences (Islam et al., 2025). Studies in several contexts have found that metallic NPs affect soil microbial populations in a concentration-dependent manner. Higher quantities, however, may impede beneficial micro-organisms important for nitrogen fixation, phosphorus solubilization and turnover of organic materials (Abbas et al., 2020). One of the major challenges in agricultural nanotechnology is to reduce the unintended effects on non-target organisms. Although nanomaterials are commonly engineered to inhibit pathogens, they could also affect beneficial microorganisms, insects, earthworms, pollinators, aquatic species and wildlife (Sadhu et al., 2026).

Repeated use of nano-enabled agricultural goods can cause the buildup of NPs in agroecosystems that impact soil, plant tissues, sediments, water bodies, and food chains, which may pose a danger to environmental sustainability. The accumulation is a function of the frequency of application, the rates of degradation, the mobility in the environment and the biological interactions. Metallic NPs are more persistent than biodegradable ones (Feng et al., 2026). The fast development of nanotechnology poses a challenge to the existing regulatory framework that was established for traditional insecticides and agrochemicals, as the special features of NPs are often not considered in the current regulations. An effective regulation requires a complete risk assessment over the whole life cycle of nanomaterials including hazard identification and exposure assessments (Kumari et al., 2023).

The effective deployment of nano-enabled plant disease control technology is not only based on the scientific performance but also on the public perception, economic feasibility and commercial acceptance. But the public concerns on environmental safety, food security and human health can hinder the acceptance of nano-based agricultural products despite the potential benefits of nanotechnology. Regulatory approval, scalability, production costs, formulation stability, and intellectual property considerations are among the numerous practical challenges associated with commercialization (Keykhasaber et al., 2026).

Major biosafety concerns and regulatory considerations associated with agricultural nanomaterials used for plant disease management. Effective risk assessment, environmental monitoring, and the development of biodegradable, microbiome-compatible nanomaterials are essential for ensuring safe and sustainable deployment in agroecosystems.

Nanotechnology is a promising approach for disrupting the virulence and communication of bacteria for a sustainable management of plant diseases. Research on nanomaterials’ effect on QS, biofilm formation and other virulence factors has progressed. Still, the large-scale implementation is challenged by scientific, technological, ecological, and regulatory hurdles, due to the reliance on lab results from simplified systems. Uncertainty about efficacy and safety is created by field conditions, microbial diversity and climate variability (Mim et al., 2025).

One of the major limitations of the present research is the lack of field studies to evaluate the nano-enabled anti-virulence technologies under actual agricultural conditions. Most studies have been conducted *in vitro*, in greenhouses, or in controlled growth chambers, where researchers have minimized environmental variability. In addition, field conditions impose many factors that can influence nanomaterial behavior including temperature variations, rainfall events, soil heterogeneity, UV radiation, interactions with organic matter, complexity of microbial community, and crop management practices. The evidence is largely available from laboratory and greenhouse studies. Large-scale field studies over several seasons and locations are rare. Environmental factors such as soil composition, UV radiation, precipitation, temperature changes and native microbial communities can strongly affect the behavior, fate and biological activity of the nanoparticles, emphasizing the need to establish standardized procedures for their evaluation in the field (Johnston et al., 2020; Abram et al., 2025).

Biological responses can be substantially different due to differences in particle size, shape, surface chemistry, synthesis methods, coating materials, and formulation strategies. For example, two silver nanoparticle formulations with similar chemical compositions may have significantly different effects on bacterial communication, biofilm formation, and plant responses because of differences in physicochemical characteristics (Barabadi et al., 2021).

One of the biggest hurdles in nano-enabled plant disease research is the lack of broadly acknowledged evaluation procedures. The present research is based on different experimental designs, measurement methodologies, strains of pathogens and assessment criteria and hence it is difficult to compare them. For example, biofilm inhibition may be measured with various staining techniques, imaging methods or genetic markers, which may result in discordant data amongst laboratories. Similarly, the assessment of QS disruption is generally conducted using different reporter systems and signaling molecules, which does not allow for direct comparison of the efficacy of NPs (Sravya and Kesavulu, 2026). QS inhibition by nanomaterials has been studied in several studies, however the molecular mechanisms are not well understood. Current data reveals that NPs may interfere with signal generation, signal degradation, receptor binding, gene regulation and communication networks. But the relative significance of various mechanisms is often unclear (Lahiri et al., 2021; Mohanta et al., 2023; Hu et al., 2024).

The use of agricultural nanomaterials remains one of the most important unsolved issues, namely the long-term ecological effects. Possible issues are alteration of microbial community structure, disturbance of beneficial symbioses, changes in nutrient cycling, soil accumulation, aquatic contamination, food chain transfer and evolutionary responses in microbial populations (Zhang et al., 2026). Despite tremendous scientific advances, major economic barriers hinder commercialization of nano-enabled anti-virulence technology. Often, the production of very specialized nanomaterials demands complicated manufacturing procedures, expensive raw materials and rigorous quality-control standards. Nano-enabled solutions need to be cost-effective, easy to use, scalable, stable during storage, regulatory compliant and perform consistently in the field to promote their wider use in agriculture (Rodriguez-Seijo et al., 2025).

Fundamental questions on nanoparticle anti-virulence remain, despite progress. How nanoparticle features affect efficacy compared with chemical composition is poorly understood. How environmental factors, such as pH and microbial diversity, affect nanoparticle-pathogen interactions is not known. In addition, the long-term evolutionary effects of the exposure of bacteria to nanoparticles are little understood. In the end, the absence of unified standards for evaluating nano-enabled anti-virulence in plant pathosystems makes it difficult to compare different investigations (Bahader et al., 2024). Addressing these gaps is crucial for translating laboratory findings to real-world applications.

There is a large variation in reported efficacy of nanoparticles between studies. Although many studies show a significant reduction in quorum sensing and biofilm formation, other studies show only moderate effects. These inconsistencies are probably due to differences in nanoparticle size, surface modifications, synthesis methods, bacterial strains, inoculum concentration, exposure time, culture medium and environmental factors rather than opposite biological processes. Thus, standardized experimental procedures will be important in increasing comparability across studies.

## 9 Future perspectives

The future of plant disease management will shift from broad-spectrum antimicrobial interventions towards precision-based strategies that selectively target pathogen virulence and communication systems (Wizrah, 2026). Unprecedented opportunities for the development of highly selective and environmentally sustainable crop protection systems have been opened by nanotechnology, synthetic biology, AI, molecular diagnostics and microbiome engineering. Future strategies will be more focused on the manipulation of pathogen behaviors, disruption of communication networks, enhancement of plant immunity and maintenance of beneficial microbial communities instead of the indiscriminate elimination of pathogens.

### 9.1 Precision anti-virulence nanotechnology

Targeted anti-virulence nanotechnology is specifically designed to inhibit pathogen virulence while minimizing effects on beneficial microorganisms and non-target species (Sahu and Satapathy, 2026). Promising nanomaterials may detect pathogen-specific molecular markers and deliver anti-virulence agents directly to sites of infection, thus enhancing treatment efficacy and supporting microbiome compatibility.

### 9.2 Stimuli-responsive smart nanomaterials

Stimuli-responsive nanomaterials release bioactive substances only in response to environmental or pathogen signals, such as pH, temperature, enzymes from pathogens, or quorum-sensing molecules. Such systems may offer increased therapeutic efficacy, reduced chemical use and lowered environmental exposure and thus are attractive candidates for future disease control (Xu et al., 2022).

### 9.3 AI-assisted design of anti-virulence nanoplatforms

AI-based methods can speed up the design and optimization of anti-virulence nanomaterials by predicting the relationship between the physical and chemical properties of nanoparticles, pathogen biology, agro-ecological responses, and environmental factors. Machine learning can improve nanoparticle size, surface chemistry, release properties, and targeting accuracy, and also help predict diseases and suggest custom treatments (Hassan et al., 2023; Tripathy et al., 2024).

### 9.4 Nano-biosensors for early detection of virulence signals

Nano-biosensors are highly sensitive in detecting molecules from pathogens before any visible symptoms of disease appear. Emerging detection platforms for quorum sensing molecules, DSFs, toxins, effector proteins, and pathogen-specific nucleic acids can be integrated with drones, wireless monitoring systems, and IoT technologies for real-time disease monitoring and rapid intervention (Masood et al., 2025).

### 9.5 Integration with biological control agents

The combination of nanotechnology with biological control has the potential to improve the survival, colonization and persistence of beneficial microorganisms in field environments. The application of nanocarriers has the potential to lead to the development of complementary strategies for sustainable disease management through protection of microbial inoculants, improved quorum-quenching strategies and rhizosphere establishment to facilitate microbial delivery (Lawal et al., 2025).

### 9.6 Climate-resilient disease management strategies

Plant diseases caused by bacteria are on the rise, with increased frequency and severity due to climate change. Nano-enabled anti-virulence strategies such as the stress-responsive formulations and the environmentally adaptive delivery systems can help to enhance crop resilience and improve disease control under variable climatic conditions (Adil et al., 2026).

### 9.7 Toward sustainable nano-enabled plant protection systems

Future plant protection systems will likely integrate anti-virulence nanomaterials, smart delivery platforms, biological control agents, decision-support systems based on artificial intelligence, nano-biosensors and microbiome engineering into coordinated management strategies. These integrated approaches can maintain beneficial microbial diversity and ecosystem functions, and allow for continuous disease monitoring, specific intervention, adaptive responses and long-term control of bacterial diseases. With the advancement in scientific knowledge and regulatory systems, nanotechnology-enabled anti-virulence technologies are poised to transform the management of plant diseases. They depart from the conventional strategy of eradicating broad-spectrum pathogens and instead aim to manipulate the pathogen behaviors at the molecular level. This approach decreases reliance on traditional pesticides, curbs resistance development, and fosters sustainable agricultural production and global food security (Agha et al., 2025; Malook et al., 2026).

## 10 Conclusions

Bacterial infections are a big threat to worldwide agriculture and novel approaches are needed to traditional disease management techniques. Nano-facilitated anti-virulence techniques are a paradigm shift from killing pathogens to reducing virulence by interfering with quorum sensing, biofilm formation and other critical pathogenic processes, which may lower the selection pressure for antimicrobial resistance. Nanomaterials have versatile properties that allow accurate and adjustable measures that can enhance crop protection with minimal impact on beneficial plant-associated microbiomes. While much progress has been achieved in the laboratory, the broad use of this technology would need extensive field validation, detailed biosafety assessments and the establishment of stringent regulatory frameworks. Future improvements should include mechanism-based nanoparticle design, particle size optimization, surface chemistry, catalytic efficiency, and functionalization based on pathogen-specific pathogenicity techniques. The application of nanotechnology with AI, precision agriculture and smart delivery systems could improve the efficacy, safety and sustainability of disease control. Collectively, these developments provide a promising platform for translation of nano-enabled anti-virulence strategies from experimental investigations into feasible eco-friendly crop protection approaches for sustainable agriculture and global food security. The development of nano-enabled anti-virulence technologies needs to be addressed regarding efficacy, long-term environmental safety, microbiome compatibility and robust field validation to ensure sustainable use in agriculture.

## References

[B1] Abbas Q. Yousaf B. Ullah H. Ali M. U. Ok Y. S. Rinklebe J. (2020). Environmental transformation and nano-toxicity of engineered nano-particles (ENPs) in aquatic and terrestrial organisms. Crit. Rev. Environ. Sci. Technol. 50, 2523–2581. doi: 10.1080/10643389.2019.170572137339054

[B2] Abdallah E. M. Alhudhaibi A. M. Hussaini I. M. Sulaiman A. N. (2026). Harnessing biogenic nanoparticles for combating antibiotic resistance: green synthesis, mechanistic insights, and biotechnological applications. Front. Bioeng. Biotechnol. 14, 1752199. doi: 10.3389/fbioe.2026.1752199 41969534 PMC13062274

[B3] Abdelgadir A. Adnan M. Patel M. Saxena J. Alam M. J. Alshahrani M. M. . (2024). Probiotic Lactobacillus salivarius mediated synthesis of silver nanoparticles (AgNPs-LS): A sustainable approach and multifaceted biomedical application. Heliyon 10, e37987. doi: 10.1016/j.heliyon.2024.e37987 39347420 PMC11437860

[B4] Abdullah A. S. Moffat C. S. Lopez-Ruiz F. J. Gibberd M. R. Hamblin J. Zerihun A. (2017). Host–multi-pathogen warfare: pathogen interactions in co-infected plants. Front. Plant Sci. 8, 1806. doi: 10.3389/fpls.2017.01806 29118773 PMC5660990

[B5] Abdulraheem M. I. Moshood A. Y. Zang Y. Mawof A. Zhang Y. Raghavan V. . (2025). Nanoparticles in food and agriculture: an overview of research progress, prospects and current knowledge. Food Biophys. 20, 61. doi: 10.1007/s11483-025-09953-y30311153

[B6] Abram S.-L. Tavernaro I. Johnston L. Zou S. Resch-Genger U. (2025). Nanoscale reference and test materials for the validation of characterization methods for engineered nanomaterials—current state, limitations, and needs. Anal. Bioanal. Chem. 417, 2405–2425. doi: 10.1007/s00216-024-05719-6 39754617 PMC12003566

[B7] Adil M. Gul I. Lu S. Lu H. Tao Y. (2026). Nanotechnology for climate-resilient agriculture: Advancing water efficiency, crop resilience, and sustainable food security. Water Air Soil pollut. 237, 653. doi: 10.1007/s11270-026-09325-330311153

[B8] Adu O. T. Mohamed F. Naidoo Y. Adu T. S. Chenia H. Dewir Y. H. . (2022). Green synthesis of silver nanoparticles from Diospyros villosa extracts and evaluation of antioxidant, antimicrobial and anti-quorum sensing potential. Plants 11, 2514. doi: 10.3390/plants11192514 36235380 PMC9573728

[B9] Afrasiabi S. Partoazar A. (2024). Targeting bacterial biofilm-related genes with nanoparticle-based strategies. Front. Microbiol. 15, 1387114. doi: 10.3389/fmicb.2024.1387114 38841057 PMC11150612

[B10] Afrasiabi S. Partoazar A. Goudarzi R. Dehpour A. R. (2025). Carbon‐based nanomaterials alter the behavior and gene expression patterns of bacteria. J. Basic Microbiol. 65, e2400545. doi: 10.1002/jobm.202400545 39895035

[B11] Afzal M. Tan X. Fang M. L. Zeng D. Huang M. Chen X. . (2025). Chitosan-silver nanoparticles and Bacillus tequilensis modulate antioxidant pathways and microbiome dynamics for sheath blight resistance in rice. Plant Stress 17, 100917. doi: 10.1016/j.stress.2025.10091742574925

[B12] Agha A. S. A. Al-Samydai A. Aburjai T. (2025). New frontiers in CRISPR: Addressing antimicrobial resistance with Cas9, Cas12, Cas13, and Cas14. Heliyon 11, e42013. doi: 10.1016/j.heliyon.2025.e42013 39906792 PMC11791237

[B13] Ahmad F. Ahmad S. (2024). “ NPs and soil microorganisms interactions in crop management-current status and future prospects,” in Industrial Applications of Soil Microbes: Volume 4. (Sharjah, United Arab Emirates: Bentham Science Publishers), 361–403. doi: 10.2174/9789815124996124040021

[B14] Ahmed T. Noman M. Gardea-Torresdey J. L. White J. C. Li B. (2023). Dynamic interplay between nano-enabled agrochemicals and the plant-associated microbiome. Trends Plant Sci. 28, 1310–1325. doi: 10.1016/j.tplants.2023.06.001 37453924

[B15] Ahsan A. Thomas N. Barnes T. J. Subramaniam S. Loh T. C. Joyce P. . (2024). Lipid nanocarriers-enabled delivery of antibiotics and antimicrobial adjuvants to overcome bacterial biofilms. Pharmaceutics 16, 396. doi: 10.3390/pharmaceutics16030396 38543290 PMC10975566

[B16] Alavi M. Jabari E. Jabbari E. (2021). Functionalized carbon-based nanomaterials and quantum dots with antibacterial activity: a review. Expert Rev. Anti-infective Ther. 19, 35–44. doi: 10.1080/14787210.2020.1810569 32791928

[B17] Algadi H. Alhoot M. A. Al-Maleki A. R. Purwitasari N. (2024). Effects of metal and metal oxide nanoparticles against biofilm-forming bacteria: a systematic review. J. Microbiol. Biotechnol. 34, 1748. doi: 10.4014/jmb.2403.03029 39099204 PMC11473618

[B18] Almeida D. P. D. Azevedo P. Z. Rodrigues R. C. Lima E. D. P. R. Stringueta P. C. Campelo P. H. . (2026). Nanoemulsion-based delivery of essential oils for controlling foodborne pathogens and spoilage microorganisms. Micro 6 (2), 33. doi: 10.3390/micro6020033

[B19] Al-Ouqaili M. T. Ahmad A. Jwair N. A. Al-Marzooq F. (2025). Harnessing bacterial immunity: CRISPR-Cas system as a versatile tool in combating pathogens and revolutionizing medicine. Front. Cell. Infect. Microbiol. 15, 1588446. doi: 10.3389/fcimb.2025.1588446 40521034 PMC12162490

[B20] Anandan K. Vittal R. R. (2025). Quorum quenching strategies of endophytic Bacillus thuringiensis KMCL07 against soft rot pathogen Pectobacterium carotovorum subsp. carotovorum. Microb. Pathogen. 200, 107356. doi: 10.1016/j.micpath.2025.107356 39921045

[B21] Arif M. (2024a). Antagonistic potential of Fusarium oxysporum as an endophyte isolated from Horse-chestnut tree in the management of Rhizoctonia solani under in-vitro conditions. Harran Tarım ve Gıda Bilimleri Dergisi 28, 550–563. doi: 10.29050/harranziraat.1524993

[B22] Arif M. (2024b). Unraveling the diversity and spatial distribution of soil-borne fungal mycobiomes with response to environmental parameters, cropping schemes and cropping seasons. J. Agric. Biotechnol. 5, 19–32. doi: 10.58728/joinabt.1486927

[B23] Arif M. (2025). Microbial sentinels in plant tissues: isolation and profiling of fungal and bacterial endophytes from diverse ecosystems of Sakarya Province. Harran Tarım ve Gıda Bilimleri Dergisi 29, 618–637. doi: 10.29050/harranziraat.1736913

[B24] Arif M. Fawaz M. S. Zuan A. T. K. Shah R. U. Ullah R. Elshehawi A. M. . (2021). The impact of different biochars on Stemphylium leaf blight (SLB) suppression and productivity of onion (Allium cepa L.). J. King Saud Univ. Sci. 33, 101575. doi: 10.1016/j.jksus.2021.10157542574925

[B25] Arif M. Islam S. U. Adnan M. Anwar M. Ali H. Wu Z. (2018). Recent progress on gene silencing/suppression by virus-derived small interfering RNAs in rice viruses especially Rice grassy stunt virus. Microb. Pathogen. 125, 210–218. doi: 10.1016/j.micpath.2018.09.021 30243549

[B26] Asif M. Xie X. Zhao Z. (2025). Regulation and inhibition of type III secretion systems in plant pathogenic bacteria. Phytopathol. Res. 7, 28. doi: 10.1186/s42483-024-00304-238164791

[B27] Awadelkareem A. M. Al-Shammari E. Elkhalifa A. O. Adnan M. Siddiqui A. J. Patel M. . (2022). Biosynthesized silver nanoparticles from Eruca sativa miller leaf extract exhibits antibacterial, antioxidant, anti-quorum-sensing, antibiofilm, and anti-metastatic activities. Antibiotics 11, 853. doi: 10.3390/antibiotics11070853 35884107 PMC9311509

[B28] Awadelkareem A. M. Siddiqui A. J. Noumi E. Ashraf S. A. Hadi S. Snoussi M. . (2023). Biosynthesized silver nanoparticles derived from probiotic Lactobacillus rhamnosus (AgNPs-LR) targeting biofilm formation and quorum sensing-mediated virulence factors. Antibiotics 12, 986. doi: 10.3390/antibiotics12060986 37370305 PMC10294857

[B29] Ayanda O. S. Mmuoegbulam A. O. Okezie O. Durumin Iya N. I. Mohammed S. A. E. James P. H. . (2024). Recent progress in carbon-based nanomaterials: critical review. J. Nanopart. Res. 26, 106. doi: 10.1007/s11051-024-06006-230311153

[B30] Azeem K. Fatima S. Ali A. Ubaid A. Husain F. M. Abid M. (2025). Biochemistry of bacterial biofilm: Insights into antibiotic resistance mechanisms and therapeutic intervention. Life. 15, 49. doi: 10.3390/life15010049 39859989 PMC11767195

[B31] Bahader A. M. Alghatis A. A. Sabbagh F. M. A. Sabbagh H. H. Almutairi F. A. Almutayri M. A. . (2024). Role of nanotechnology in antibiotic drug delivery: Review study on the advancement of transforming infection management in critical care, challenges and future directions. J. Int. Crisis Risk Communication Res. 7, 2355. doi: 10.63278/jicrcr.vi.1586

[B32] Banerjee R. (2025). Tiny but mighty: Small RNAs—The micromanagers of bacterial survival, virulence, and host–pathogen interactions. Non-coding RNA 11, 36. doi: 10.3390/ncrna11030036 40407594 PMC12101431

[B33] Barabadi H. Mojab F. Vahidi H. Marashi B. Talank N. Hosseini O. . (2021). Green synthesis, characterization, antibacterial and biofilm inhibitory activity of silver nanoparticles compared to commercial silver nanoparticles. Inorg. Chem. Commun. 129, 108647. doi: 10.1016/j.inoche.2021.10864742574925

[B34] Barton I. S. Eagan J. L. Nieves-Otero P. A. Reynolds I. P. Platt T. G. Fuqua C. (2021). Co-dependent and interdigitated: dual quorum sensing systems regulate conjugative transfer of the Ti plasmid and the At megaplasmid in Agrobacterium tumefaciens 15955. Front. Microbiol. 11, 605896. doi: 10.3389/fmicb.2020.605896 33552018 PMC7856919

[B35] Beig B. Niazi M. B. K. Sher F. Jahan Z. Malik U. S. Khan M. D. . (2022). Nanotechnology-based controlled release of sustainable fertilizers. A review. Environ. Chem. Lett. 20, 2709–2726. doi: 10.1007/s10311-022-01409-w30311153

[B36] Bhardwaj I. Bhardwaj N. Kumar V. Kumari S. Dulta K. Aman J. . (2025). Synergistic effects of Bacillus cereus Mn‐5 PGPR‐derived silver oxide nanoparticles on tomato plant growth, stress resilience and nutritional enhancement. J. Sustain. Agric. Environ. 4, e70090. doi: 10.1002/sae2.7009042587375

[B37] Bhuiyan M. N. I. (2025). Biofilm, resistance, and quorum sensing: The triple threat in bacterial pathogenesis. Microbe 9, 100578. doi: 10.1016/j.microb.2025.100578 42574925

[B38] Boban T. Nadar S. Tauro S. (2023). Breaking down bacterial communication: a review of quorum quenching agents. Future J. Pharm. Sci. 9, 77. doi: 10.1186/s43094-023-00526-938164791

[B39] Braylé P. Pinelli E. Gauthier L. Mouchet F. Barret M. (2022). Graphene-based nanomaterials and microbial communities: a review of their interactions, from ecotoxicology to bioprocess engineering perspectives. Environ. Sci. Nano 9, 3725–3741. doi: 10.1039/D2EN00547

[B40] Cao Y. Turk K. Bibi N. Ghafoor A. Ahmed N. Azmat M. . (2025). Nanoparticles as catalysts of agricultural revolution: enhancing crop tolerance to abiotic stress: a review. Front. Plant Sci. 15, 1510482. doi: 10.3389/fpls.2024.1510482 39898270 PMC11782286

[B41] Carezzano M. E. Paletti Rovey M. F. Cappellari L. D. R. Gallarato L. A. Bogino P. Oliva M. D. L. M. . (2023). Biofilm-forming ability of phytopathogenic bacteria: a review of its involvement in plant stress. Plants 12, 2207. doi: 10.3390/plants12112207 37299186 PMC10255566

[B42] Chachar Z. Xue X. Fang J. Chen M. Jiarui C. Chen W. . (2025). Key mechanisms of plant-Ralstonia solanacearum interaction in bacterial wilt pathogenesis. Front. Microbiol. 16, 1521422. doi: 10.3389/fmicb.2025.1521422 40547798 PMC12179194

[B43] Chakraborty M. Hasanuzzaman M. Rahman M. Khan M. A. R. Bhowmik P. Mahmud N. U. . (2020). Mechanism of plant growth promotion and disease suppression by chitosan biopolymer. Agriculture 10, 624. doi: 10.3390/agriculture1012062430654563

[B44] Chen Y. Gao Y. Huang Y. Jin Q. Ji J. (2023). Inhibiting quorum sensing by active targeted pH-sensitive nanoparticles for enhanced antibiotic therapy of biofilm-associated bacterial infections. ACS Nano 17, 10019–10032. doi: 10.1021/acsnano.2c12151 37234036

[B45] Chen H. Singh H. Bhardwaj N. Bhardwaj S. K. Khatri M. Kim K.-H. . (2022). An exploration on the toxicity mechanisms of phytotoxins and their potential utilities. Crit. Rev. Environ. Sci. Technol. 52, 395–435. doi: 10.1080/10643389.2020.182317237339054

[B46] Chen M. Xia L. Wu C. Wang Z. Ding L. Xie Y. . (2024). Microbe-material hybrids for therapeutic applications. Chem. Soc Rev. 53, 8306–8378. doi: 10.1039/d3cs00655g 39005165

[B47] Chen Y. Zhu L. Yan X. Liao Z. Teng W. Wang Y. . (2025). Significant roles of nanomaterials for enhancing disease resistance in rice: a review. Agronomy 15, 1938. doi: 10.3390/agronomy1508193830654563

[B48] Condinho M. Carvalho B. Cruz A. Pinto S. N. Arraiano C. M. Pobre V. (2023). The role of RNA regulators, quorum sensing and c‐di‐GMP in bacterial biofilm formation. FEBS Open Bio 13, 975–991. doi: 10.1002/2211-5463.13389 35234364 PMC10240345

[B49] Cucu M. A. Choudhary R. Trkulja V. Garg S. Matić S. (2025). Utilizing environmentally friendly techniques for the sustainable control of plant pathogens: A review. Agronomy 15, 1551. doi: 10.3390/agronomy1507155130654563

[B50] D’Angelo S. (2026). Micro-and nanoplastics in agroecosystems: Plant uptake, food safety, and implications for human health. Sustainability 18, 2817. doi: 10.3390/su18062817

[B51] Dabare S. Rajapaksha S. Munaweera I. (2025). Exploring the intersection of innovation management and nanotechnology in sustainable agriculture. Int. J. Innovation Sci., 1–37. doi: 10.1108/ijis-03-2025-009535579975

[B52] Das D. Ingti B. Paul P. Jamatia J. P. Ete T. Khan T. . (2025). Soil microbial dynamics in response to the impact of nanoparticles on agricultural implications. Discover Soil 2, 58. doi: 10.1007/s44378-025-00087-830311153

[B53] De Pessemier C. Vandecasteele M. Hamonts K. Van Damme D. Goormachtig S. (2026). Licensed to protect: microbial biocontrol challenges and opportunities. Trends Plant Sci. doi: 10.1016/j.tplants.2026.03.012 42014242

[B54] DeTemple M. F. Zabotina O. A. (2025). Different strategies of altering the cell wall to study the impact that these introduced changes have on plant resistance to pathogens. Front. Plant Sci. 16, 1680357. doi: 10.3389/fpls.2025.1680357 41158683 PMC12555068

[B55] Doan V. C. (2025). Rare earth elements in agroecosystems: a review of plant defenses and cascading effects on insect herbivores and pollinators. J. Plant Interact. 20, 2581395. doi: 10.1080/17429145.2025.258139537339054

[B56] Eid H. M. Almutawif Y. A. Khan N. U. (2026). Triad warfare against biofilms: disrupting quorum sensing, nanotechnology-driven precision, and host immunomodulation to conquer antimicrobial resistance. Arch. Microbiol. 208, 211. doi: 10.1007/s00203-026-04750-8 41724773

[B57] Elbasuney S. Yehia M. Ismael S. Al-Hazmi N. E. El-Sayyad G. S. Tantawy H. (2023). Potential impact of reduced graphene oxide incorporated metal oxide nanocomposites as antimicrobial, and antibiofilm agents against pathogenic microbes: Bacterial protein leakage reaction mechanism. J. Cluster Sci. 34, 823–840. doi: 10.1007/s10876-022-02255-030311153

[B58] Elbehiry A. Abalkhail A. (2025). Antimicrobial nanoparticles against superbugs: mechanistic insights, biomedical applications, and translational frontiers. Pharmaceuticals 18, 1195. doi: 10.3390/ph18081195 40872586 PMC12389178

[B59] Elmegdar S. Elkheloui R. Laktib A. Mimouni R. Hamadi F. (2024). Antibiofilm and anti-quorum sensing activities of biological nanoparticles. Curr. Appl. Sci. Technol. 24 (2), e0257479. doi: 10.55003/cast.2023.257479

[B60] Elmer W. White J. C. (2018). The future of nanotechnology in plant pathology. Annu. Rev. Phytopathol. 56, 111–133. doi: 10.1146/annurev-phyto-080417-050108 30149792

[B61] Elshaer S. L. Shaaban M. I. (2021). Inhibition of quorum sensing and virulence factors of Pseudomonas aeruginosa by biologically synthesized gold and selenium nanoparticles. Antibiotics 10, 1461. doi: 10.3390/antibiotics10121461 34943673 PMC8698379

[B62] El-Shetehy M. Moradi A. Maceroni M. Reinhardt D. Petri-Fink A. Rothen-Rutishauser B. . (2021). Silica nanoparticles enhance disease resistance in Arabidopsis plants. Nat. Nanotechnol. 16, 344–353. doi: 10.1038/s41565-020-00812-0 33318639 PMC7610738

[B63] Farrukh M. Munawar A. Nawaz Z. Hussain N. Hafeez A. B. Szweda P. (2025). Antibiotic resistance and preventive strategies in foodborne pathogenic bacteria: a comprehensive review. Food Sci. Biotechnol. 34, 2101. doi: 10.1007/s10068-024-01767-x 40351726 PMC12064539

[B64] Fathi Qarachal J. Alizadeh M. (2025). Harnessing precision agriculture and nanotechnology for sustainable farming: a review. Nanotechnol. Environ. Eng. 10, 37. doi: 10.1007/s41204-025-00429-530311153

[B65] Feng J. Adeel M. Rui Y. (2026). Advancing nano-enabled agriculture from precision application to environmental risk and sustainability assessment. Curr. pollut. Rep. 12, 5. doi: 10.1007/s40726-026-00397-730311153

[B66] Feng Z. Guo Y. Zhang Y. Zhang A. Jia M. Yin J. . (2024). Nanozymes: a bibliometrics review. J. Nanobiotechnol. 22, 704. doi: 10.1186/s12951-024-02907-5 39538291 PMC11562681

[B67] Filipić B. Ušjak D. Rambaher M. H. Oljacic S. Milenković M. T. (2024). Evaluation of novel compounds as anti-bacterial or anti-virulence agents. Front. Cell. Infect. Microbiol. 14, 1370062. 10.3389/fcimb.2024.1370062 PMC10951914 38510964

[B68] Fincheira P. Hoffmann N. Tortella G. Ruiz A. Cornejo P. Diez M. C. . (2023). Eco-efficient systems based on nanocarriers for the controlled release of fertilizers and pesticides: Toward smart agriculture. Nanomaterials 13, 1978. doi: 10.3390/nano13131978 37446494 PMC10343595

[B69] Fleitas Martínez O. Cardoso M. H. Ribeiro S. M. Franco O. L. (2019). Recent advances in anti-virulence therapeutic strategies with a focus on dismantling bacterial membrane microdomains, toxin neutralization, quorum-sensing interference and biofilm inhibition. Front. Cell. Infect. Microbiol. 9, 74. doi: 10.3389/fcimb.2019.00074 31001485 PMC6454102

[B70] Flemming H.-C. van Hullebusch E. D. Little B. J. Neu T. R. Nielsen P. H. Seviour T. . (2025). Microbial extracellular polymeric substances in the environment, technology and medicine. Nat. Rev. Microbiol. 23, 87–105. doi: 10.1038/s41579-024-01098-y 39333414

[B71] Flemming H.-C. van Hullebusch E. D. Neu T. R. Nielsen P. H. Seviour T. Stoodley P. . (2023). The biofilm matrix: multitasking in a shared space. Nat. Rev. Microbiol. 21, 70–86. doi: 10.1038/s41579-022-00791-0 36127518

[B72] Gao M. Delgado-Baquerizo M. Xiong C. Sáez-Sandino T. Wang J. Liang J. . (2026). Dominance and natural suppression of bacterial plant pathogens across global soils. Nat. Commun. doi: 10.1038/s41467-026-70233-5 41786734 PMC13125666

[B73] Garg D. Sridhar K. Stephen Inbaraj B. Chawla P. Tripathi M. Sharma M. (2023). Nano-biofertilizer formulations for agriculture: A systematic review on recent advances and prospective applications. Bioengineering 10, 1010. doi: 10.3390/bioengineering10091010 37760112 PMC10525541

[B74] Geszke-Moritz M. Moritz M. (2024). Biodegradable polymeric nanoparticle-based drug delivery systems: comprehensive overview, perspectives and challenges. Polymers 16, 2536. doi: 10.3390/polym16172536 39274168 PMC11397980

[B75] Giese B. Klaessig F. Park B. Kaegi R. Steinfeldt M. Wigger H. . (2018). Risks, release and concentrations of engineered nanomaterial in the environment. Sci. Rep. 8, 1565. doi: 10.1038/s41598-018-19275-4 29371617 PMC5785520

[B76] Gomez-Villalba L. S. Salcines C. Fort R. (2023). Application of inorganic nanomaterials in cultural heritage conservation, risk of toxicity, and preventive measures. Nanomaterials 13, 1454. doi: 10.3390/nano13091454 37176999 PMC10180185

[B77] Grenz K. Chia K.-S. Turley E. K. Tyszka A. S. Atkinson R. E. Reeves J. . (2025). A necrotizing toxin enables Pseudomonas syringae infection across evolutionarily divergent plants. Cell. Host Microbe 33, 20–29. e25. doi: 10.1016/j.chom.2024.11.014 39706183

[B78] Gudepu R. Kyatham R. Ediga N. D. Penta G. Bathula R. Alam M. M. . (2026). The multifaceted mechanistic actions of antimicrobial nanoformulations: Overcoming resistance and enhancing efficacy. Pharmaceutics 18, 423. doi: 10.3390/pharmaceutics18040423 42076075 PMC13118510

[B79] Guo S. Mao B. Tang X. Zhang Q. Zhao J. Chen W. . (2026). Autoinducer 2 as a universal language in microbial consortia: decoding molecular mechanisms, ecological impacts, and application. Gut Microbes 18, 2615494. doi: 10.1080/19490976.2026.2615494 41574816 PMC12834153

[B80] Hajipour M. J. Saei A. A. Walker E. D. Conley B. Omidi Y. Lee K. B. . (2021). Nanotechnology for targeted detection and removal of bacteria: opportunities and challenges. Adv. Sci. 8, 2100556. doi: 10.1002/advs.202100556 34558234 PMC8564466

[B81] Hashem A. H. Ibrahim N. A. Badr B. M. Selim M. T. Elkady F. M. Issa N. I. . (2025). Exploring the biological activities of the Origanum majorana essential oil nanoemulsion: antimicrobial, antibiofilm, antioxidant and anticancer activities. RSC Adv. 15, 46870–46881. doi: 10.1039/d5ra06064h 41323708 PMC12658965

[B82] Hassan S.-D. Almaliki M. N. S. Hussein Z. A. Albehadili H. M. Rabeea Banoon S. Abboodi A. . (2023). Development of nanotechnology by artificial intelligence: a comprehensive review. J. Nanostruct. 13, 915–932. doi: 10.22052/JNS.2023.04.002

[B83] Hayat M. K. Arif M. Turan F. Mahmud M. N. Batool R. Cengiz R. . (2026). Assessing microplastic translocation from contaminated agricultural soils to food crops. Discover Soil 3, 124. doi: 10.1007/s44378-026-00281-230311153

[B84] Hejabi F. Abbaszadeh M. S. Taji S. O’Neill A. Farjadian F. Doroudian M. (2022). Nanocarriers: A novel strategy for the delivery of CRISPR/Cas systems. Front. Chem. 10, 957572. doi: 10.3389/fchem.2022.957572 36092658 PMC9450496

[B85] Hetta H. F. Ramadan Y. N. Rashed Z. I. Alharbi A. A. Alsharef S. Alkindy T. T. . (2024). Quorum sensing inhibitors: an alternative strategy to win the battle against multidrug-resistant (MDR) bacteria. Molecules 29, 3466. doi: 10.3390/molecules29153466 39124871 PMC11313800

[B86] Hu C. He G. Yang Y. Wang N. Zhang Y. Su Y. . (2024). Nanomaterials regulate bacterial quorum sensing: Applications, mechanisms, and optimization strategies. Adv. Sci. 11, 2306070. doi: 10.1002/advs.202306070 38350718 PMC11022734

[B87] Hussain S. Arif A. Shah M. R. (2026). Targeted drug delivery: designing nanocarriers for improved therapeutic action. Chem. Commun. doi: 10.1039/d5cc07306e 41729230

[B88] Hussain A. Noman A. Arif M. Farooq S. Khan M. I. Cheng P. . (2021). A basic helix-loop-helix transcription factor CabHLH113 positively regulate pepper immunity against Ralstonia solanacearum. Microb. Pathogen. 156, 104909. doi: 10.1016/j.micpath.2021.104909 33964418

[B89] Hussain M. Shakoor N. Adeel M. Ahmad M. A. Zhou H. Zhang Z. . (2023). Nano-enabled plant microbiome engineering for disease resistance. Nano Today 48, 101752. doi: 10.1016/j.nantod.2023.10175242574925

[B90] Ijaz M. Sattar A. Sher A. Ul-Allah S. Mansha M. Z. Khan K. A. . (2021). Sulfur application combined with planomicrobium sp. Strain MSSA-10 and farmyard manure biochar helps in the management of charcoal rot disease in sunflower (Helianthus annuus L.). Sustainability 13, 8535. doi: 10.3390/su1315853530654563

[B91] Iqbal M. (2026). Microbial biocontrol agents and the rhizosphere microbiome: integrating ecological function and climate resilience in sustainable agriculture. Front. Microbiol. 17, 1771649. doi: 10.3389/fmicb.2026.1771649 41834876 PMC12979447

[B92] Islam S. (2025). Toxicity and transport of nanoparticles in agriculture: effects of size, coating, and aging. Front. Nanotechnol. 7, 1622228. doi: 10.3389/fnano.2025.1622228

[B93] Islam A. N. Uddin M. N. Ridwan A. Neon A. K. Rab M. F. (2025). Engineering nanoparticles (ENPs) in aquatic environments and soil-plant ecosystems: transformation, toxicity, and environmental challenges. Front. Soil Sci. 5, 1705689. doi: 10.3389/fsoil.2025.1705689

[B94] Jacobowski A. C. Boleti A. P. A. Cruz M. V. Santos K. F. D. P. de Andrade L. R. M. Frihling B. E. F. . (2025). Combating antimicrobial resistance: Innovative strategies using peptides, nanotechnology, phages, quorum sensing interference, and CRISPR-Cas systems. Pharmaceuticals 18, 1119. doi: 10.3390/ph18081119 40872511 PMC12389514

[B95] Jiang Y. Meng F. Ge Z. Zhou Y. Fan Z. Du J. (2024). Bioinspired peptide/polyamino acid assemblies as quorum sensing inhibitors for the treatment of bacterial infections. J. Mater. Chem. B 12, 11596–11610. doi: 10.1039/d4tb01685h 39436377

[B96] Johnston L. J. Gonzalez-Rojano N. Wilkinson K. J. Xing B. (2020). Key challenges for evaluation of the safety of engineered nanomaterials. NanoImpact 18, 100219. doi: 10.1016/j.impact.2020.10021942574925

[B97] Joshi H. (2025). Exploring the efficacy of green nanoparticles in enhancing plant defense: a mechanistic investigation into immune response activation. J. Nanopart. Res. 27, 29. doi: 10.1007/s11051-025-06226-030311153

[B98] Joubert I. A. Geppert M. Ess S. Nestelbacher R. Gadermaier G. Duschl A. . (2020). Public perception and knowledge on nanotechnology: A study based on a citizen science approach. NanoImpact 17, 100201. doi: 10.1016/j.impact.2019.10020142574925

[B99] Juszczuk-Kubiak E. (2024). Molecular aspects of the functioning of pathogenic bacteria biofilm based on quorum sensing (QS) signal-response system and innovative non-antibiotic strategies for their elimination. Int. J. Mol. Sci. 25, 2655. doi: 10.3390/ijms25052655 38473900 PMC10931677

[B100] Kalelkar P. P. Riddick M. García A. J. (2022). Biomaterial-based antimicrobial therapies for the treatment of bacterial infections. Nat. Rev. Mater. 7, 39–54. doi: 10.1038/s41578-021-00362-4 35330939 PMC8938918

[B101] Kalia A. Abd-Elsalam K. A. Kuca K. (2020). Zinc-based nanomaterials for diagnosis and management of plant diseases: Ecological safety and future prospects. J. Fungi 6, 222. doi: 10.3390/jof6040222 33066193 PMC7711620

[B102] Kalu C. M. Ogugua U. V. Udeh E. L. Otun S. Oladipo A. O. Lebelo S. L. . (2026). Applications of nanoparticles in plant disease identification and control for sustainable crop production. Discover Nano 21, 15. doi: 10.1186/s11671-026-04440-w 41581110 PMC12832606

[B103] Kapeleka J. A. Mwema M. F. (2024). State of nano pesticides application in smallholder agriculture production systems: human and environmental exposure risk perspectives. Heliyon 10, e39225. doi: 10.1016/j.heliyon.2024.e39225 39492887 PMC11530829

[B104] Karthikeyan A. Tabassum N. Jeong G.-J. Javaid A. Mani A. K. Kim T.-H. . (2025). Alleviation of mycobacterial infection by impairing motility and biofilm formation via natural and synthetic molecules. World J. Microbiol. Biotechnol. 41, 113. doi: 10.1007/s11274-025-04322-w 40148661

[B105] Kaur S. Chand U. Sharma R. Chamoli S. Kumar P. (2026). Review of nanomaterials, natural products, and their composites to combat antimicrobial resistance. ACS Appl. Nano Mater. doi: 10.1021/acsanm.6c00232

[B106] Kemppinen J. Pollmeier M. Ehonen S. Brosché M. Sierla M. (2025). Water immunity overrides stomatal immunity in plant resistance to Pseudomonas syringae. Plant Physiol. 198, kiaf127. doi: 10.1093/plphys/kiaf127 40173409 PMC12063527

[B107] Keykhasaber M. Tzelepis G. Rafiei V. (2026). Converging technologies for next-generation plant protection: an integrated framework for fungal disease management. Front. Agron. 8, 1760693. doi: 10.3389/fagro.2026.1760693

[B108] Khalifa H. O. Oreiby A. Mohammed T. Abdelhamid M. A. Sholkamy E. N. Hashem H. . (2025). Silver nanoparticles as next-generation antimicrobial agents: mechanisms, challenges, and innovations against multidrug-resistant bacteria. Front. Cell. Infect. Microbiol. 15, 1599113. doi: 10.3389/fcimb.2025.1599113 40895297 PMC12390974

[B109] Khan A. Ashiq B. Qadri T. A. Khattak W. A. Anas M. Khan R. A. . (2025). Nanotechnology solutions for advancing sustainable development goals (SDGs): Integrating nanomaterials for global sustainability. BioNanoScience 15, 1–22. doi: 10.1007/s12668-025-02214-930311153

[B110] Kumar S. Paliya B. S. Singh B. N. (2022). Superior inhibition of virulence and biofilm formation of Pseudomonas aeruginosa PAO1 by phyto-synthesized silver nanoparticles through anti-quorum sensing activity. Microb. Pathogen. 170, 105678. doi: 10.1016/j.micpath.2022.105678 35820580

[B111] Kumari R. Suman K. Karmakar S. Mishra V. Lakra S. G. Saurav G. K. . (2023). Regulation and safety measures for nanotechnology-based agri-products. Front. Genome Ed. 5, 1200987. doi: 10.3389/fgeed.2023.1200987 37415849 PMC10320728

[B112] Lahiri D. Nag M. Sheikh H. I. Sarkar T. Edinur H. A. Pati S. . (2021). Microbiologically-synthesized nanoparticles and their role in silencing the biofilm signaling cascade. Front. Microbiol. 12, 636588. doi: 10.3389/fmicb.2021.636588 33717030 PMC7947885

[B113] Lawal H. Gaddafi M. S. Jamiu A. M. Edo G. S. Fremah O. G. El-Yakub A. U. . (2025). Biocontrol and nanotechnology strategies for postharvest disease management in fruits and vegetables: a comprehensive review. Foods 14, 2782. doi: 10.3390/foods14162782 40870695 PMC12385622

[B114] Lee P. Lin X. Khan F. Bennett A. E. Winter J. O. (2022). Translating controlled release systems from biomedicine to agriculture. Front. Biomater. Sci. 1, 1011877. doi: 10.3389/fbiom.2022.1011877

[B115] Letizia M. Diggle S. P. Whiteley M. (2025). Pseudomonas aeruginosa: ecology, evolution, pathogenesis and antimicrobial susceptibility. Nat. Rev. Microbiol. 23, 701–717. doi: 10.1038/s41579-025-01193-8 40442328 PMC13064840

[B116] Liu F. Hu M. Zhang Z. Xue Y. Chen S. Hu A. . (2022). Dickeya manipulates multiple quorum sensing systems to control virulence and collective behaviors. Front. Plant Sci. 13, 838125. doi: 10.3389/fpls.2022.838125 35211146 PMC8860905

[B117] Liu D. Lu Y. Li Z. Pang X. Gao X. (2025). Quorum sensing: not just a Bridge between bacteria. Microbiol. Open 14, e70016. doi: 10.1002/mbo3.70016 40159675 PMC11955508

[B118] Ma G. Zou Y. Wang S. Guo X. Li Z. Li H. . (2025). Advancing sustainable agriculture with mesoporous nanomaterials for smart pesticide delivery. J. Agric. Food. Chem. 73, 18480–18496. doi: 10.1021/acs.jafc.5c04278 40680262

[B119] Makhlouf Z. Ali A. A. Al-Sayah M. H. (2023). Liposomes-based drug delivery systems of anti-biofilm agents to combat bacterial biofilm formation. Antibiotics 12, 875. doi: 10.3390/antibiotics12050875 37237778 PMC10215698

[B120] Malešević M. Jovčić B. (2025). Targeting Gram-Negative bacterial biofilm with innovative therapies: communication silencing strategies. Future Pharmacol. 5, 35. doi: 10.3390/futurepharmacol5030035

[B121] Malook M. B. Ijaz M. Ijaz R. Shang J. Lv L. Ahmed T. . (2026). Nanotechnology-based strategies for sustainable management of bacterial plant diseases: mechanisms, applications, and future directions. Environ. Sci. Nano 13, 723–747. doi: 10.1039/d5en00936g

[B122] Manisha Y. Srinivasan M. Jobichen C. Rosenshine I. Sivaraman J. (2024). Sensing for survival: specialised regulatory mechanisms of type III secretion systems in Gram‐negative pathogens. Biol. Rev. 99, 837–863. doi: 10.1111/brv.13047 38217090

[B123] Masood R. Khan M. Rahim A. (2025). A review of recent advancement in biosensors development and their applications. Trends Med. Biol. Sci. 1, 33–65.

[B124] Masood H. A. Qi Y. Zahid M. K. Li Z. Ahmad S. Lv J.-M. . (2024). Recent advances in nano-enabled immunomodulation for enhancing plant resilience against phytopathogens. Front. Plant Sci. 15, 1445786. doi: 10.3389/fpls.2024.1445786 39170781 PMC11336869

[B125] McCourt K. M. Cochran J. Abdelbasir S. M. Carraway E. R. Tzeng T.-R. Tsyusko O. V. . (2022). Potential environmental and health implications from the scaled-up production and disposal of nanomaterials used in biosensors. Biosensors 12, 1082. doi: 10.3390/bios12121082 36551049 PMC9775545

[B126] Mehta M. Bhushan I. (2024). Potential of biosynthesized titanium dioxide nanoparticles towards wastewater treatment and antimicrobial activity. 3 Biotech. 14, 66. doi: 10.1007/s13205-024-03915-w 38351910 PMC10859355

[B127] Meng Q. An X. Hu W. Ma M. Chen Z. Wei G. . (2026). Nanopriming with silicon quantum dots strengthens wheat drought tolerance through physiological regulation and microbial functions. J. Agric. Food. Chem. 74, 5989–6001. doi: 10.1021/acs.jafc.5c11900 41698031

[B128] Mgadi K. Ndaba B. Roopnarain A. Rama H. Adeleke R. (2024). Nanoparticle applications in agriculture: overview and response of plant-associated microorganisms. Front. Microbiol. 15, 1354440. doi: 10.3389/fmicb.2024.1354440 38511012 PMC10951078

[B129] Mim J. J. Rakib S. Akter S. Nisha J. R. Khan S. Rahman S. . (2025). The emerging role of machine learning in nanomaterials research: applications, challenges, and future directions. J. Nanopart. Res. 27, 1–51. doi: 10.1007/s11051-025-06472-230311153

[B130] Mohanta Y. K. Chakrabartty I. Mishra A. K. Chopra H. Mahanta S. Avula S. K. . (2023). Nanotechnology in combating biofilm: a smart and promising therapeutic strategy. Front. Microbiol. 13, 1028086. doi: 10.3389/fmicb.2022.1028086 36938129 PMC10020670

[B131] Mujtaba M. Wang D. Carvalho L. B. Oliveira J. L. Espirito Santo Pereira A. D. Sharif R. . (2021). Nanocarrier-mediated delivery of miRNA, RNAi, and CRISPR-Cas for plant protection: current trends and future directions. ACS Agric. Sci. Technol. 1, 417–435. doi: 10.1021/acsagscitech.1c00146

[B132] Mukarram M. Ali J. Dadkhah-Aghdash H. Kurjak D. Kačík F. Ďurkovič J. (2023). Chitosan-induced biotic stress tolerance and crosstalk with phytohormones, antioxidants, and other signalling molecules. Front. Plant Sci. 14, 1217822. doi: 10.3389/fpls.2023.1217822 37538057 PMC10394624

[B133] Mukherjee S. Bassler B. L. (2019). Bacterial quorum sensing in complex and dynamically changing environments. Nat. Rev. Microbiol. 17, 371–382. doi: 10.1038/s41579-019-0186-5 30944413 PMC6615036

[B134] Muñoz I. V. Sarrocco S. Malfatti L. Baroncelli R. Vannacci G. (2019). CRISPR-Cas for fungal genome editing: a new tool for the management of plant diseases. Front. Plant Sci. 10, 135. doi: 10.3389/fpls.2019.00135 PMC6384228 30828340

[B135] Naga N. G. El-Badan D. E. Ghanem K. M. Shaaban M. I. (2023). It is the time for quorum sensing inhibition as alternative strategy of antimicrobial therapy. Cell Commun. Signaling 21, 1–14. doi: 10.1186/s12964-023-01154-9 37316831 PMC10265836

[B136] Nguyen P. V. Darnetty Lina E. C. Duong N. V. Ho P. T. Huýnh D. B. (2025). Nanotechnology for managing rice blast disease: a comprehensive review. J. Nanotheranostics 6, 23. doi: 10.3390/jnt603002330654563

[B137] Nielipinski M. Pietrzyk-Brzezinska A. J. Wlodawer A. Sekula B. (2023). Structural analysis and molecular substrate recognition properties of Arabidopsis thaliana ornithine transcarbamylase, the molecular target of phaseolotoxin produced by Pseudomonas syringae. Front. Plant Sci. 14, 1297956. doi: 10.3389/fpls.2023.1297956 38179474 PMC10765591

[B138] Niroula D. Bhandari K. Sundin G. W. Joshi J. R. (2026). Virulence regulation and lifestyle transitions: the role of c‐di‐GMP and two‐component systems in Erwinia amylovora and their evolutionary context within Enterobacterales. Mol. Plant Pathol. 27, e70228. doi: 10.1111/mpp.70228 PMC12910134 41699890

[B139] Nithyanand P. Boya B. R. Lee J. H. Lee J. (2025). Polymicrobial biofilms: interkingdom interactions, resistance and therapeutic strategies. Microb. Biotechnol. 18, e70218. doi: 10.1111/1751-7915.70218 40847578 PMC12373980

[B140] Oluwole O. A. Campos E. V. R. de Oliveira J. L. Fraceto L. F. (2025). Integrating the nano–phyto–micro triad for climate-resilient and sustainable agriculture. ACS Omega 10, 53702–53721. doi: 10.1021/acsomega.5c07151 41280856 PMC12631361

[B141] Pagano M. Lunetta E. Belli F. Mocarli G. Cocozza C. Cacciotti I. (2025). Advancements in agricultural nanotechnology: an updated review. Plants 14, 2939. doi: 10.3390/plants14182939 41012090 PMC12473408

[B142] Pal P. Prakash O. Parveen A. Singh A. K. Gupta R. Sarangi P. K. . (2025). Nanoparticle-driven plant signaling for advancing stress resilience and agricultural productivity-a review. J. Nanopart. Res. 27, 258. doi: 10.1007/s11051-025-06449-130311153

[B143] Papaneophytou C. (2026). Phytochemical quorum-sensing inhibitors against bacterial pathogens: mechanisms of action and translational challenges. Curr. Issues Mol. Biol. 48, 214. doi: 10.3390/cimb48020214 41751475 PMC12939507

[B144] Parvin N. Joo S. W. Mandal T. K. (2025). Nanomaterial-based strategies to combat antibiotic resistance: mechanisms and applications. Antibiotics 14, 207. doi: 10.3390/antibiotics14020207 40001450 PMC11852044

[B145] Patil N. P. Shevate S. N. (2025). Targeting acyl homoserine lactone regulated quorum sensing by quorum quenching enzymes. Biologia 80, 2479–2501. doi: 10.1007/s11756-025-02006-230311153

[B146] Pellosi D. S. Paiva G. S. Vital V. G. Mendes A. L. Santos N. G. Kuriki F. K. . (2025). Harnessing copper nanoparticles for antimicrobial applications: advances and challenges. Antibiotics 14, 1170. doi: 10.3390/antibiotics14111170 41301665 PMC12649138

[B147] Pietroiusti A. Stockmann‐Juvala H. Lucaroni F. Savolainen K. (2018). Nanomaterial exposure, toxicity, and impact on human health. Wiley Interdiscip. Reviews: Nanomedicine Nanobiotechnology 10, e1513. doi: 10.1002/wnan.1513 29473695

[B148] Piperno A. Scala A. Mazzaglia A. Neri G. Pennisi R. Sciortino M. T. . (2018). Cellular signaling pathways activated by functional graphene nanomaterials. Int. J. Mol. Sci. 19, 3365. doi: 10.3390/ijms19113365 30373263 PMC6274994

[B149] Porcari A. Borsella E. Benighaus C. Grieger K. Isigonis P. Chakravarty S. . (2019). From risk perception to risk governance in nanotechnology: a multi-stakeholder study. J. Nanopart. Res. 21, 245. doi: 10.1007/s11051-019-4689-930311153

[B150] Qu Y. Zou Y. Wang G. Zhang Y. Yu Q. (2024). Disruption of communication: recent advances in antibiofilm materials with anti-quorum sensing properties. ACS Appl. Materials Interfaces 16, 13353–13383. doi: 10.1021/acsami.4c01428 38462699

[B151] Quintarelli V. Ben Hassine M. Radicetti E. Stazi S. R. Bratti A. Allevato E. . (2024). Advances in nanotechnology for sustainable agriculture: A review of climate change mitigation. Sustainability 16, 9280. doi: 10.20944/preprints202406.1570.v1

[B152] Qureshi K. A. Fahmy N. A. Parvez A. Almahasheer H. Permatasari D. Jaremko M. . (2026). Biofilms and antimicrobial resistance: Mechanisms, clinical implications, and emerging interventions. Chem. Biodivers. 23, e01351. doi: 10.1002/cbdv.202501351 41417517

[B153] Raghuvanshi N. Kumar C. Parmar K. Tomar A. Kumar V. Singh N. . (2026). The nano-reactor and the microbial enzyme activation: Unmasking the dualistic power of nanoparticles for sustainable soil health. J. Soil Sci. Plant Nutr., 102–138. doi: 10.1007/s42729-026-03240-630311153

[B154] Rajkhowa S. Hussain S. Z. Agarwal M. Zaheen A. Al-Hussain S. A. Zaki M. E. (2024). Advancing antibiotic-resistant microbe combat: Nanocarrier-based systems in combination therapy targeting quorum sensing. Pharmaceutics 16, 1160. doi: 10.3390/pharmaceutics16091160 39339197 PMC11434747

[B155] Ramburrun P. Varughese T. P. Choonara Y. E. (2025). Nanotheranostics in periodontitis: Bridging diagnosis and therapy through smart integrated nanosystems. J. Nanotheranostics 6, 31. doi: 10.3390/jnt604003130654563

[B156] Ramesh K. Ruf A. Robatzek S. (2026). Catch me if you can: How Xylella fastidiosa thrives in the xylem. Annu. Rev. Phytopathol. 64. doi: 10.1146/annurev-phyto-011325-110907 42285547

[B157] Ramôa A. M. Campos F. Moreira L. Teixeira C. Leiro V. Gomes P. . (2023). Antimicrobial peptide-grafted PLGA-PEG nanoparticles to fight bacterial wound infections. Biomater. Sci. 11, 499–508. 10.1039/d2bm01127a 36458466

[B158] Ranjbar S. Emamjomeh A. Sharifi F. Zarepour A. Aghaabbasi K. Dehshahri A. . (2023). Lipid-based delivery systems for flavonoids and flavonolignans: Liposomes, nanoemulsions, and solid lipid nanoparticles. Pharmaceutics 15, 1944. doi: 10.3390/pharmaceutics15071944 37514130 PMC10383758

[B159] Rasmussen K. Sayre P. Kobe A. Gonzalez M. Rauscher H. (2025). 25 years of research and regulation: Is nanotechnology safe to commercialize? Front. Toxicol. 7, 1629813. doi: 10.3389/ftox.2025.1629813 40708971 PMC12286942

[B160] Raya J. Montagut E.-J. Marco M.-P. (2025). Analysing the integrated quorum sensing (iqs) system and its potential role in Pseudomonas aeruginosa pathogenesis. Front. Cell. Infect. Microbiol. 15, 1575421. doi: 10.3389/fcimb.2025.1575421 40568711 PMC12188538

[B161] Rehman M. Tahir N. Sohail M. F. Qadri M. U. Duarte S. O. Brandão P. . (2024). Lipid-based nanoformulations for drug delivery: An ongoing perspective. Pharmaceutics 16, 1376. doi: 10.3390/pharmaceutics16111376 39598500 PMC11597327

[B162] Riseh R. S. Fathi F. Vazvani M. G. Tarkka M. T. (2025). Plant colonization by biocontrol bacteria and improved plant health: A review. Front. Bioscience-Landmark 30, 23223. doi: 10.31083/fbl23223 39862070

[B163] Rodrigues A. S. Batista J. G. Rodrigues M.Á. Thipe V. C. Minarini L. A. Lopes P. S. . (2024). Advances in silver nanoparticles: A comprehensive review on their potential as antimicrobial agents and their mechanisms of action elucidated by proteomics. Front. Microbiol. 15, 1440065. doi: 10.3389/fmicb.2024.1440065 39149204 PMC11325591

[B164] Rodriguez-Seijo A. Santas-Miguel V. Arenas-Lago D. Arias-Estevez M. Perez-Rodriguez P. (2025). Use of nanotechnology for safe agriculture and food production: Challenges and limitations. Pedosphere 35, 20–32. doi: 10.1016/j.pedsph.2024.09.00542574925

[B165] Romulo A. Suryoprabowo S. Setiarto R. H. B. Guo Y. (2025). Carbon dots as multifunctional nanomaterials: A review on antimicrobial activities and fluorescence-based microbial detection. Molecules 30, 3969. doi: 10.3390/molecules30193969 41097389 PMC12526252

[B166] Roy D. Nag M. Bhattacharya D. Lahiri D. (2023). Microbiologically synthesized nanoparticles in inhibition of quorum sensing. Natural Products, 102–138. doi: 10.1201/9781003300557-6

[B167] Ryan R. P. Vorhölter F.-J. Potnis N. Jones J. B. Van Sluys M.-A. Bogdanove A. J. . (2011). Pathogenomics of Xanthomonas: Understanding bacterium–plant interactions. Nat. Rev. Microbiol. 9, 344–355. doi: 10.1038/nrmicro2558 21478901

[B168] Saati-Santamaría Z. Pérez-Mendoza D. Khashi u Rahman M. de Sousa B. F. S. Montero-Calasanz M. D. C. Rey L. . (2026). Evolutionary mechanisms underlying bacterial adaptation to the plant environment. FEMS Microbiol. Rev. 50, fuag005. doi: 10.1093/femsre/fuag005 41671169 PMC12949522

[B169] Sadhu A. Ghosh S. Mandal A. H. Chatterjee S. Saha N. C. Biswas J. K. . (2026). Toxicological profile of titanium dioxide nanoparticles: Properties, production and potential toxic effects on non-target aquatic organisms. Water Air Soil pollut. 237, 537. doi: 10.1007/s11270-026-09165-130311153

[B170] Saeki E. K. Martins H. M. Camargo L. C. D. Anversa L. Tavares E. R. Yamada-Ogatta S. F. . (2022). Effect of biogenic silver nanoparticles on the quorum-sensing system of Pseudomonas aeruginosa PAO1 and PA14. Microorganisms 10, 1755. doi: 10.3390/microorganisms10091755 36144357 PMC9504124

[B171] Sahli C. Moya S. E. Lomas J. S. Gravier-Pelletier C. Briandet R. Hémadi M. (2022). Recent advances in nanotechnology for eradicating bacterial biofilm. Theranostics 12, 2383. doi: 10.7150/thno.67296 35265216 PMC8899562

[B172] Sahu P. Satapathy T. (2026). Beyond new pills: Integrative strategies to overcome multidrug-resistant bacteria. Arch. Microbiol. 208, 276. doi: 10.1007/s00203-026-04817-6 41843109

[B173] Saini S. Yadav R. Das R. Singh C. (2026). Enzyme therapy as a promising strategy to overcome multi-drug resistance in pathogens: Current advances, key challenges, and future directions. RSC Adv. 16, 5578–5605. doi: 10.1039/d5ra06932g 41602185 PMC12833820

[B174] Salam A. Khan S. M. Afridi M. S. Khan A. R. Zeeshan M. Rehman M. . (2026). Recent advances in nanomaterial-based approaches for plant disease management and early detection. J. Agric. Food. Chem. 74, 7322–7349. doi: 10.1021/acs.jafc.5c12329 41746974

[B175] Saleh R. M. Hassan O. M. (2024). Antibacterial, antibiofilm, and quorum quenching properties of biogenic chitosan silver nanoparticles against Staphylococcus aureus. BioNanoScience 14, 4456–4468. doi: 10.1007/s12668-024-01573-z30311153

[B176] Sargazi S. Arshad R. Ghamari R. Rahdar A. Bakhshi A. Karkan S. F. . (2022). siRNA‐based nanotherapeutics as emerging modalities for immune‐mediated diseases: A preliminary review. Cell Biol. Int. 46, 1320–1344. doi: 10.1002/cbin.11841 35830711 PMC9543380

[B177] Sato Y. Note Y. Maeki M. Kaji N. Baba Y. Tokeshi M. . (2016). Elucidation of the physicochemical properties and potency of siRNA-loaded small-sized lipid nanoparticles for siRNA delivery. J. Controlled Release 229, 48–57. doi: 10.1016/j.jconrel.2016.03.019 26995758

[B178] Scalia A. C. Najmi Z. (2025). Targeting bacterial biofilms on medical implants: Current and emerging approaches. Antibiotics 14, 802. doi: 10.3390/antibiotics14080802 40867996 PMC12382947

[B179] Sedighi O. Bednarke B. Sherriff H. Doiron A. L. (2024). Nanoparticle-based strategies for managing biofilm infections in wounds: A comprehensive review. ACS Omega 9, 27853–27871. doi: 10.1021/acsomega.4c02343 38973924 PMC11223148

[B180] Selim H. M. R. M. Gomaa F. A. M. Alshahrani M. Y. Aboshanab K. M. (2025). Role of CRISPR-Cas system as a new approach in fighting the antimicrobial resistance of bacterial and viral pathogens. Infect. Dis. Immun. 5, 127–137. doi: 10.1097/id9.0000000000000149 42328613 PMC13277551

[B181] Sellami H. Akinyemi M. O. Gdoura-Ben Amor M. Onwudiwe D. C. Mthiyane D. M. (2025). Structural and optical characterization of TiO2 nanoparticles synthesized using Globularia alypum leaf extract and the antibacterial properties. Discover Appl. Sci. 7, 834. doi: 10.1007/s42452-025-07521-030311153

[B182] Sengupta A. Bhattacharya D. Lahiri D. Nag M. Dey A. Ray S. (2025). Gold and silver nanoparticles as potent quorum quenchers: A critical review. BioNanoScience 15, 73. doi: 10.1007/s12668-024-01721-530311153

[B183] Shafi Z. Shahid M. Singh A. Raj G. B. Rasool A. (2026). Engineering Trichoderma-mediated plant defense against bacterial phytopathogens: Micro-and nanobiotechnological strategies. AIMS Microbiol. 12, 27. doi: 10.3934/microbiol.2026002 41938783 PMC13047323

[B184] Shah S. Gaikwad S. Nagar S. Kulshrestha S. Vaidya V. Nawani N. . (2019). Biofilm inhibition and anti-quorum sensing activity of phytosynthesized silver nanoparticles against the nosocomial pathogen Pseudomonas aeruginosa. Biofouling 35, 34–49. doi: 10.1080/08927014.2018.1563686 30727758

[B185] Shariati A. Chegini Z. Ghaznavi-Rad E. Zare E. N. Hosseini S. M. (2022). PLGA-based nanoplatforms in drug delivery for inhibition and destruction of microbial biofilm. Front. Cell. Infect. Microbiol. 12, 926363. doi: 10.3389/fcimb.2022.926363 35800390 PMC9253276

[B186] Sharma A. Gupta A. Devi B. (2023). Current trends in management of bacterial pathogens infecting plants. Antonie van Leeuwenhoek 116, 303–326. doi: 10.1007/s10482-023-01809-0 36683073

[B187] Sikdar R. Elias M. (2020). Quorum quenching enzymes and their effects on virulence, biofilm, and microbiomes: A review of recent advances. Expert Rev. Anti-Infective Ther. 18, 1221–1233. doi: 10.1080/14787210.2020.1794815 32749905 PMC7705441

[B188] Sil M. Goswami A. Mukherjee N. (2026). Effects of mesoporous silica nanoparticles on seedling growth of Vigna radiata and glycolytic metabolism in Bacillus coagulans. ACS Agric. Sci. Technol. 6, 561–574. doi: 10.1021/acsagscitech.5c00794

[B189] Singh H. Balusamy S. R. Sukweenadhi J. Saravanan M. Aruchamy M. Mijakovic I. . (2025b). Smart hybrid nanomaterials for chronic infections: Microbiome-responsive and sustainable therapeutic platforms. J. Nanobiotechnol. 23, 698. doi: 10.1186/s12951-025-03712-4 41184906 PMC12581504

[B190] Singh B. Dahiya M. Kumar V. Ayyagari A. Chaudhari D. N. Ahire J. J. . (2025a). Biofilm and antimicrobial resistance: Mechanisms, implications, and emerging solutions. Microbiol. Res. 16, 183. doi: 10.3390/microbiolres1608018330654563

[B191] Singh S. Prasad S. M. Bashri G. (2023). Fate and toxicity of nanoparticles in aquatic systems. Acta Geochim. 42, 63–76. doi: 10.1007/s11631-022-00572-930311153

[B192] Singh A. A. Singh A. K. (2025). Role of bacterial quorum sensing in plant growth promotion. World J. Microbiol. Biotechnol. 41, 18. doi: 10.1007/s11274-024-04232-3 39724256

[B193] Skurková D. Zhukouskaya H. Buziková M. Mahun A. Kanizsová L. Vetrík M. . (2025). Catalytic degradation of N-acyl-homoserine lactone using a copper complex of a TACN derivative: Implications for quorum sensing interference. Dalton Trans. 54, 16106–16120. doi: 10.1039/D5DT016 41084744

[B194] Soares C. A. Gois I. B. de Andrade Nascimento L. F. Gois L. S. Santos J. S. Blank A. F. . (2026). Croton grewioides Baill essential oil reduces biofilm formation and the virulence of Xanthomonas campestris pv. campestris. Biocatal. Agric. Biotechnol. 72, 103913. doi: 10.1016/j.bcab.2025.10391342574925

[B195] Sodhi G. K. Wijesekara T. Kumawat K. C. Adhikari P. Joshi K. Singh S. . (2025). Nanomaterials–plants–microbes interaction: Plant growth promotion and stress mitigation. Front. Microbiol. 15, 1516794. doi: 10.3389/fmicb.2024.1516794 39881995 PMC11774922

[B196] Sompiyachoke K. Elias M. H. (2024). Engineering quorum quenching acylases with improved kinetic and biochemical properties. Protein Sci. 33, e4954. doi: 10.1002/pro.4954 38520282 PMC10960309

[B197] Song C. Pan H. Wang Y. Liao R. Arif M. Wang H. . (2025a). Penicillium restrictum reshapes root exudates and inhibits Peucedanum praeruptorum bolting through field inoculation and co‐cultivation. Plant Pathol. 74, 2035–2053.

[B198] Song C. Shah I. H. Ashraf G. A. Arif M. Manzoor M. A. (2025b). Zinc oxide nanoparticle alleviates the inhibition of Dendrobium huoshanense photosynthesis by cadmium through enhancing antioxidant enzyme system. Phyton-International J. Exp. Bot. 94, 3427–3451. doi: 10.32604/phyton.2025.070778

[B199] Sravya B. Kesavulu M. M. (2026). Harnessing plant-derived nanoparticles for sustainable agriculture: Applications, challenges, and prospects. Nanochemistry Res. 11, 210–233.

[B200] Stabryla L. M. Johnston K. A. Diemler N. A. Cooper V. S. Millstone J. E. Haig S.-J. . (2021). Role of bacterial motility in differential resistance mechanisms of silver nanoparticles and silver ions. Nat. Nanotechnol. 16, 996–1003. doi: 10.1038/s41565-021-00929-w 34155383

[B201] Su T. Zhang L. Shen J. Qian D. Guo Y. Li Z. (2025). Beyond pathogenicity: applications of the type III secretion system (T3SS) of Pseudomonas aeruginosa. Front. Microbiol. 16, 1663945. doi: 10.3389/fmicb.2025.1663945 40964673 PMC12439673

[B202] Subramoni S. Nathoo N. Klimov E. Yuan Z.-C. (2014). Agrobacterium tumefaciens responses to plant-derived signaling molecules. Front. Plant Sci. 5, 322. doi: 10.3389/fpls.2014.00322 25071805 PMC4086400

[B203] Suchánková L. Kvítek L. Kolář M. Panáček A. (2026). Emerging strategies of bacterial adaptation mechanisms to silver and metal oxide nanomaterials. FEMS Microbiol. Rev. 50, fuaf060. 10.1093/femsre/fuaf060 PMC12757747 41363705

[B204] Sun Y. Li J. Huang J. Li S. Li Y. Lu B. . (2025). Architecture of genome-wide transcriptional regulatory network reveals dynamic functions and evolutionary trajectories in Pseudomonas syringae. Elife 13, RP96172. doi: 10.7554/elife.96172.1 PMC11957545 40162990

[B205] Takita K. Someya N. Morohoshi T. (2025). Distribution and functional analysis of two types of quorum sensing gene pairs, glaI1/glaR1 and glaI2/glaR2, in Burkholderia gladioli. FEMS Microbiol. Lett. 372, fnae117. 10.1093/femsle/fnae117 PMC11753530 39762131

[B206] Tam Y. Y. C. Chen S. Cullis P. R. (2013). Advances in lipid nanoparticles for siRNA delivery. Pharmaceutics 5, 498–507. doi: 10.3390/pharmaceutics5030498 24300520 PMC3836621

[B207] Touati A. Ibrahim N. A. Tighilt L. Idres T. (2025). Anti-QS strategies against Pseudomonas aeruginosa infections. Microorganisms 13, 1838. doi: 10.3390/microorganisms13081838 40871342 PMC12388730

[B208] Tripathy A. Patne A. Y. Mohapatra S. Mohapatra S. S. (2024). Convergence of nanotechnology and machine learning: the state of the art, challenges, and perspectives. Int. J. Mol. Sci. 25, 12368. doi: 10.3390/ijms252212368 39596433 PMC11594285

[B209] Tu Z. Wang Y. Qian X. Chen J. Li L. Wang T. . (2026). Plasmonic magnetic nanoparticles-enabled universal enrichment, photothermal lysis, and duplex CRISPR detection of bacteria in urine samples. Biosens. Bioelectron. 302, 118518. doi: 10.1016/j.bios.2026.118518 41707427

[B210] Urbauer E. Gräwe A. Chamas A. Papenfort K. (2026). Engineering quorum sensing: new directions for antimicrobial therapy. Trends Microbiol. doi: 10.1016/j.tim.2026.05.006 42185145

[B211] Utari P. D. Vogel J. Quax W. J. (2017). Deciphering physiological functions of AHL quorum quenching acylases. Front. Microbiol. 8, 1123. doi: 10.3389/fmicb.2017.01123 28674525 PMC5474475

[B212] Vadakkan K. (2026). A comprehensive review on various mechanisms of quorum sensing inhibition by gold nanoparticles. Curr. Microbiol. 83, 314. doi: 10.1007/s00284-026-04917-y 41999422

[B213] van Gent M. E. Ali M. Nibbering P. H. Kłodzińska S. N. (2021). Current advances in lipid and polymeric antimicrobial peptide delivery systems and coatings for the prevention and treatment of bacterial infections. Pharmaceutics 13, 1840. doi: 10.3390/pharmaceutics13111840 34834254 PMC8618997

[B214] Vu Thanh C. Gooding J. J. Kah M. (2025). Learning lessons from nano-medicine to improve the design and performances of nano-agrochemicals. Nat. Commun. 16, 2306. doi: 10.1038/s41467-025-57650-8 40055366 PMC11889108

[B215] Wang L. Huang J. Chen S. Su X. Zhang X. Wang L. . (2024). Endogenous cell wall degrading enzyme LytD is important for the biocontrol activity of Bacillus subtilis. Front. Plant Sci. 15, 1381018. doi: 10.3389/fpls.2024.1381018 38660441 PMC11039861

[B216] Wang X. Liu M. Yu C. Li J. Zhou X. (2023). Biofilm formation: mechanistic insights and therapeutic targets. Mol. Biomed. 4, 49. doi: 10.1186/s43556-023-00164-w 38097907 PMC10721784

[B217] Wang L. Ning C. Pan T. Cai K. (2022). Role of silica nanoparticles in abiotic and biotic stress tolerance in plants: a review. Int. J. Mol. Sci. 23, 1947. doi: 10.3390/ijms23041947 35216062 PMC8872483

[B218] Whiteley M. Diggle S. P. Greenberg E. P. (2017). Progress in and promise of bacterial quorum sensing research. Nature 551, 313–320. doi: 10.1038/nature24624 29144467 PMC5870893

[B219] Wijesinghe G. Nobbs A. Bandara H. (2025). The diffusible signaling factor family in microbial signaling: a current perspective. Crit. Rev. Microbiol. 51, 975–990. doi: 10.1080/1040841x.2025.2457670 39868787

[B220] Wizrah M. S. (2026). CRISPR-Cas systems as next-generation antimicrobials: a systemic review of mechanisms, delivery strategies, and translational challenges. Front. Microbiol. 17, 1747931. doi: 10.3389/fmicb.2026.1747931 42199538 PMC13199036

[B221] Wu S. Jin Z. Wang P. Song R. Song B. (2026). Chemical management of phytopathogenic bacteria: emerging compounds, molecular targets and outlook. Chem. Soc Rev. doi: 10.1039/d5cs00248f 41431378

[B222] Wu J.-M. Liu Y. Han H.-Y. Song Z.-Y. (2023). Recent advances in endogenous and exogenous stimuli-responsive nanoplatforms for bacterial infection treatment. Biomed. Eng. Commun. 2, 2–23. doi: 10.53388/bmec2023002

[B223] Wypij M. Trzcińska-Wencel J. Golińska P. Avila-Quezada G. D. Ingle A. P. Rai M. (2023). The strategic applications of natural polymer nanocomposites in food packaging and agriculture: Chances, challenges, and consumers’ perception. Front. Chem. 10, 1106230. doi: 10.3389/fchem.2022.1106230 36704616 PMC9871319

[B224] Xiao S. Lu X. Gou L. Li J. Ma Y. Liu J. . (2019). Graphene oxide as antibacterial sensitizer: Mechanically disturbed cell membrane for enhanced poration efficiency of melittin. Carbon 149, 248–256. doi: 10.1016/j.carbon.2019.04.06742574925

[B225] Xin X.-F. Kvitko B. He S. Y. (2018). Pseudomonas syringae: what it takes to be a pathogen. Nat. Rev. Microbiol. 16, 316–328. doi: 10.1038/nrmicro.2018.17 29479077 PMC5972017

[B226] Xing Y. Jiang H. Cai L. (2025). Engineered nanotransporters for efficient RNAi delivery in plant protection applications. J. Integr. Plant Biol. 67, 1223–1245. doi: 10.1111/jipb.13887 40080402

[B227] Xiong C. Ge A.-H. Gao M. Singh B. K. (2026). Impacts of climate extremes on plant pathogens, microbiomes and plant health. Nat. Rev. Microbiol. 24, 637–653. doi: 10.1038/s41579-026-01290-2 41814018

[B228] Xu K.-Z. Vinothkanna A. Wang D. Jiang H. Xu Z. Jia A.-Q. (2026). Bacterial quorum sensing systems in cell-cell communication. FEMS Microbiol. Rev. 50, fuag020. doi: 10.1093/femsre/fuag020 42114269 PMC13220807

[B229] Xu T. Wang Y. Aytac Z. Zuverza-Mena N. Zhao Z. Hu X. . (2022). Enhancing agrichemical delivery and plant development with biopolymer-based stimuli responsive core–shell nanostructures. ACS Nano 16, 6034–6048. doi: 10.1021/acsnano.1c11490 35404588

[B230] Yadav A. Yadav K. (2024). Exploring the effect of engineered nanomaterials on soil microbial diversity and functions: a review. J. Environ. Nanotechnol. 13, 48–62. doi: 10.13074/jent.2024.03.241503

[B231] Yang P. Condrich A. Lu L. Scranton S. Hebner C. Sheykhhasan M. . (2024). Genetic engineering in bacteria, fungi, and oomycetes, taking advantage of CRISPR. DNA 4, 427–454. doi: 10.3390/dna404003030654563

[B232] Yarahmadi A. Najafiyan H. Yousefi M. H. Khosravi E. Shabani E. Afkhami H. . (2025). Beyond antibiotics: exploring multifaceted approaches to combat bacterial resistance in the modern era: a comprehensive review. Front. Cell. Infect. Microbiol. 15, 1493915. doi: 10.3389/fcimb.2025.1493915 40176987 PMC11962305

[B233] Yeh Y.-C. Huang T.-H. Yang S.-C. Chen C.-C. Fang J.-Y. (2020). Nano-based drug delivery or targeting to eradicate bacteria for infection mitigation: a review of recent advances. Front. Chem. 8, 286. doi: 10.3389/fchem.2020.00286 32391321 PMC7193053

[B234] Zarepour A. Venkateswaran M. R. Khosravi A. Iravani S. Zarrabi A. (2024). Bioinspired nanomaterials to combat microbial biofilm and pathogen challenges: A review. ACS Appl. Nano Mater. 7, 25287–25313. doi: 10.1021/acsanm.4c04806

[B235] Zeng S. Shakoor N. Rui Y. (2025). Nanotechnology and agricultural sustainability: a review. Nanomaterials 15, 1755. doi: 10.3390/nano15231755 41369432 PMC12693324

[B236] Zhang J. Dou T. Shen Y. Wang W. Wang L. Wu X. . (2024a). Application of nanozymes in problematic biofilm control: Progress, challenges and prospects. Front. Environ. Sci. Eng. 18, 136. doi: 10.1007/s11783-024-1896-030311153

[B237] Zhang W. Fan X. Li J. Ye T. Mishra S. Zhang L. . (2021). Exploration of the quorum-quenching mechanism in Pseudomonas nitroreducens W-7 and its potential to attenuate the virulence of Dickeya zeae EC1. Front. Microbiol. 12, 694161. doi: 10.3389/fmicb.2021.694161 34413838 PMC8369503

[B238] Zhang Y. Hussain A. Arif M. Alkahtani J. AlMunqedhi B. M. Song C. (2024b). Genetic variability for salinity tolerance of tomato (Solanum lycopersicon MILL.) genotypes determined by stress tolerance indices. J. King Saud Univ. Sci. 36, 103386. doi: 10.1016/j.jksus.2024.10338642574925

[B239] Zhang X. Kong H. Yang G. Zhu D. Luan X. He P. . (2022). Graphene-based functional hybrid membranes for antimicrobial applications: A review. Appl. Sci. 12, 4834. doi: 10.3390/app1210483430654563

[B240] Zhang T. Wang Q. Rui Y. (2025). The impact of nanomaterials on plant health: a review of exposure, toxicity, and control. Environ. Sci. Nano 12, 2965–2982. doi: 10.1039/d5en00037h

[B241] Zhang H. Zhang Z. Li J. Qin G. (2023). New strategies for biocontrol of bacterial toxins and virulence: Focusing on quorum-sensing interference and biofilm inhibition. Toxins 15, 570. doi: 10.3390/toxins15090570 37755996 PMC10536320

[B242] Zhang J. Zhang T. Rui Y. (2026). From nanomaterials to nanofertilizers: applications, ecological risks, and prospects for sustainable agriculture. Plants 15, 415. doi: 10.3390/plants15030415 41681580 PMC12899010

[B243] Zhao Z. Z. Guo L. Li X. Cheng T. Chu C. H. Zhang J. (2025). Quorum quenching by Est816: a novel approach to control Porphyromonas gingivalis pathogenicity. BMC Oral. Health 25, 1178. doi: 10.1186/s12903-025-06563-5 40671022 PMC12269267

[B244] Zhou Y. Tang L. Chen D. Li Y. Wu S. Qiao J. (2026). Analysis and engineering of quorum sensing-based communications between bacteria and fungi. Mbio 17, e03838-03825. doi: 10.1128/mbio.03838-25 41801051 PMC13059760

